# Spatial and maturity heterogeneity of tertiary lymphoid structures shapes immune microenvironment and progression in prostate cancer

**DOI:** 10.1016/j.jncc.2025.06.003

**Published:** 2025-07-24

**Authors:** Zhongyuan Wang, Qintao Ge, Ren Mo, Jiahe Lu, Xi Tian, Aihetaimujiang Anwaier, Shiqi Ye, Siqi Zhou, Weihang Guo, Chuanhai Cai, Jianfeng Yang, Hailiang Zhang, Xiaojian Qin, Dingwei Ye, Wenhao Xu

**Affiliations:** 1 Department of Urology, Fudan University Shanghai Cancer Center; Department of Oncology, Shanghai Medical College, Fudan University, Shanghai, China; 2 Shanghai Genitourinary Cancer Institute, Shanghai, China; 3 Department of Urology, Inner Mongolia People’s Hospital, Inner Mongolia Urological Institute, Hohhot, China; 4 Department of Urology, Longhua Hospital, Shanghai University of Traditional Chinese Medicine, Shanghai, China

**Keywords:** Prostate cancer, Tumor microenvironment, Tertiary lymphoid structures, High endothelial venules, Spatial multi-omics

## Abstract

**Background:**

Tertiary lymphoid structure (TLS), ectopic lymphoid aggregates formed in response to chronic inflammation, have emerged as potential prognostic biomarkers and mediators of anti-tumor immunity in various cancers. However, the heterogeneity of TLS spatial distribution, maturity, and their prognostic and immunological significance in prostate cancer (PCa) remain poorly characterized.

**Methods:**

We utilized immunohistochemistry, multispectral fluorescence immunohistochemistry (mIHC) and spatial multi-omics analyses to evaluate the heterogeneity of TLS and its relationship with immune components in the tumor microenvironment (TME). Prognostic implications were assessed in 701 PCa patients from the TCGA and Fudan University Shanghai Cancer Center cohorts. The association between TLS heterogeneity and immunoreactivity was assessed through the quantification of immune cell infiltration. CellTreck and robust cell type decomposition deconvolution algorithms were used to decipher the colocalization features of each cell, cell-cell communication and ligand-receptor features within TLS regions.

**Results:**

In PCa, TLSs were detected in approximately 20 % of patients across both cohorts, with intratumoral TLS (intra-TLS) being twice as prevalent as peritumoral TLS (peri‑TLS). Patients harboring intra-TLS exhibited significantly longer disease-free and progression-free survival. Compared to peri‑TLS, intra-TLS were more mature, characterized by increased T-effector cell infiltration, activation of interferon pathways, and the presence of follicular dendritic cell centers and B cell aggregates. Notably, compared with immature TLS, mature TLS were markedly associated with reduced PD-L1 expression, lower regulatory T cells (Tregs) infiltration, and increased high endothelial venules (HEVs) density, indicative of an immunologically active microenvironment. Spatial multi-omics analysis revealed that mature TLS exhibited enriched immune cell diversity and HEVs density, suggesting enhanced anti-tumor immunity. Furthermore, cell-cell communication analysis identified significant interactions between CCL5^+^ dendritic cells and ACKR1^+^ activated B cells within mature TLS, reflecting the enhanced capacity of mature TLS to orchestrate robust antigen presentation and B-cell-driven immune responses.

**Conclusions:**

In conclusion, this study highlights the prognostic and immunological implications of TLS heterogeneity in PCa, demonstrating that the spatial distribution and maturity of TLSs are closely linked to TME activation and improved clinical outcomes. These findings provide novel insights into the immune landscape of PCa and establish a foundation for immune-based precision stratification and therapeutic development.

## 1 Introduction

Prostate cancer (PCa) accounts for 29 % of new cancer diagnoses and 11 % of cancer-related deaths in men globally.^1^ With over 10 million individuals already diagnosed with PCa and an estimated 700,000 annual deaths by 2040, PCa represents a significant global health burden.^2^, ^3^, ^4^ While androgen deprivation therapy (ADT) is the primary treatment for metastatic PCa, the development of acquired resistance is an almost universal outcome for most patients.^5^ Additionally, emerging immune checkpoint inhibitors have demonstrated only limited anticancer activity in PCa.^6^^,^^7^ Given that PCa is a highly heterogeneous malignancy, characterized by diverse genetic phenotypes and mutation profiles,^8^^,^^9^ the identification and screening of effective biomarkers for selecting suitable candidates for immunotherapy remain a major challenge in PCa management. Consequently, the discovery of tissue biomarkers that can effectively probe the PCa microenvironment holds substantial clinical significance.

While tumor-draining lymph nodes play a critical role in tumor-targeted immunity,^10^ many solid tumors harbor tertiary lymphoid structure (TLS). Unlike primary and secondary lymphoid organs, TLS is aggregates of immune cells that form in response to chronic inflammation or persistent immune stimulation.^11^ The lack of a fibrous capsule around TLS allows direct contact between immune cells and tumor tissue, bypassing the need for antigen-presenting cells and lymphocytes to migrate between the tumor and secondary lymphoid organs, thereby enabling a rapid anti-tumor immune response.^12^ TLSs are distributed throughout the mesenchyme, tumor margins, and core in various cancers,^13^ with their formation influenced by distinct mechanisms at different spatial levels. The spatial distribution of TLSs, both within and around the tumor, as well as their degree of maturity, plays a critical role in modulating the recruitment, activation, and interactions of immune cells. Intratumoral TLS (Intra-TLS) has emerged as prognostic biomarkers that can predict and potentially enhance the efficacy of immunotherapy across multiple cancer types.^14^^,^^15^ Conversely, peritumoral TLS (peri‑TLS), although supported by limited data, may be associated with diminished immunotherapy responses and poorer patient prognosis.^16^^,^^17^ On the other hand, immature TLS, characterized by disorganized T or B cell aggregates, may be insufficient to induce robust antitumor immunity and are often associated with an immunosuppressive tumor microenvironment (TME).^18^^,^^19^ In contrast, mature TLS have been linked to improved prognosis and enhanced responses to immunotherapy in ovarian, lung, and bladder cancers. This association may be attributed to the more organized aggregation of immune cells and the induction of antibody-mediated apoptosis by follicular dendritic cells (FDCs), B cell lymphoma 6 (BCL-6) germinal center B cells, plasma cells, and T cells.^13^^,^^20^^,^^21^ Mature TLS serves as specific sites for dendritic cell (DC) local antigen presentation and contribute to the generation of tumor-targeting CD8^+^ effector memory T cells, memory B cells, and antibody-producing plasma cells, all of which arise from well-structured germinal centers.^20^^,^^22^ The presence of mature TLS can predict a stronger immune response and better prognosis, and may even independently predict the efficacy of immune checkpoint inhibitors, regardless of PD-L1 expression. This predictive value extends across different tumor types, including those typically resistant to immune checkpoint blockade (ICB) therapy, such as pancreatic cancer and sarcoma.^23^

Immune checkpoint inhibitors (ICIs) have revolutionized cancer immunotherapy, demonstrating remarkable efficacy across various tumor types where high frequencies of TLS are often correlated with improved prognosis.^23^^,^^24^ In contrast with tumors such as melanoma, renal cell carcinoma, and non-small cell lung cancer, which are highly responsive to ICIs, PCa exhibits limited sensitivity to these therapies. This resistance is likely attributable to PCa’s relatively low tumor mutational burden (TMB) and the presence of robust immunosuppressive mechanisms that dampen anti-tumor immune responses.^4^^,^^25^, ^26^, ^27^ Although the roles of B cells, plasma cells, and humoral adaptive immunity in PCa have been increasingly recognized, the precise characteristics of TLS in PCa and their functional contributions to the tumor microenvironment remain poorly understood.^28^^,^^29^ This knowledge gap underscores the need for a deeper investigation into the clinical and biological significance of TLS in PCa.

The primary aim of this study was to comprehensively characterize the heterogeneity of TLS in PCa, focusing on their spatial distribution, maturation status, and associated changes in the TME. Additionally, we investigated the prognostic significance of TLS in PCa patients and explored how TLS maturation influences immune cell infiltration and stromal composition. By elucidating these factors, this study provides valuable insights into the role of TLS in shaping the TME and their potential utility as biomarkers for patient stratification and prognosis.

## 2 Materials and methods

### 2.1 Samples collection and cohorts construction

The study cohort included 200 localized PCa patients who underwent radical prostatectomy at the Urology Department of Fudan University Shanghai Cancer Center (FUSCC, Shanghai, China) between August 2010 and December 2013, as well as 501 PCa patients from The Cancer Genome Atlas (TCGA) database (Supplementary Fig. 1). Patients were included if they met the following criteria: (1) patients diagnosed with localized prostate adenocarcinoma (confirmed via histopathological and clinical evaluation); (2) complete medical records and follow-up data; and (3) no prior treatment before surgery, ensuring that the tumor microenvironment reflects its natural, untreated state. Patients were excluded if: (1) medical records or follow-up data were incomplete; (2) the diagnosis was not prostatic acinar adenocarcinoma (e.g., small cell carcinoma, neuroendocrine prostate cancer); or (3) pathological specimens lacked adjacent normal tissue, which is critical for comparative analysis of TLS distribution. Data were collected from electronic medical records and pathological reports, including demographic information (age), clinical data (stage, regional lymph node status, biochemical recurrence status), and pathological data (histological type, Gleason score).

The TCGA cohort served as the primary dataset for transcriptomic data analysis and morphological characterization based on hematoxylin and eosin (HE) staining. To investigate the mutational landscape of PCa, copy number variation (CNV) data were retrieved using standardized procedures from the TCGA Data Portal. Somatic variants were identified and analyzed using the “maftools” R package. Oncoplots were generated to visualize mutation profiles, highlighting frequently mutated genes and their mutation frequencies in the TCGA cohort.

The clinicopathological features of the 200 PCa patients in the FUSCC cohort and the 501 patients in the TCGA cohort are summarized in Table 1. These comprehensive datasets provided a foundation for subsequent analyses, including transcriptomic profiling, TLS heterogeneity assessment, and TME characterization.

### 2.2 Hematoxylin and eosin staining and immunohistochemistry assays

For the pathological analysis of the FUSCC cohort samples, three non-consecutive 4 μm-thick sections were obtained from each tumor, with an interval of >200 μm between consecutive sections. This approach ensured representative sampling from both central and peripheral tumor regions while minimizing potential bias arising from a single-section analysis. In the TCGA cohort, tumor samples included paraffin-embedded and frozen sections, with slice thicknesses ranging from 4 to 5 µm. These sections underwent HE staining and were subsequently digitized through Whole Slide Imaging.

The digitized slides from the TCGA cohort were obtained from the GDC Data Portal (https://portal.gdc.cancer.gov/), ensuring accessibility and reproducibility of the data. All HE-stained slides were independently reviewed by two experienced pathologists for histopathological assessment. This ensured consistency and accuracy in the evaluation of tumor features and TLS localization.^30^

PCa tissues or adjacent normal prostate tissues were selected for H&E staining to assess the localization of TLS. After HE staining, the specimens showed potential TLS, and the final consecutive sections of the PCa specimens were identified by immunohistochemistry to confirm the presence of definitive TLS. The TLSs were identified as lymphocyte aggregates, which had histological characteristics similar to lymphoid tissues with B cells (CD20, ab64088, Abcam), T cells (CD3, ab16669, Abcam), FDCs (CD21, ab7290, Abcam) and germinal center (GC) cells (CD23, ab16702, Abcam). Identification of TLS positivity from PCa carriers involved the identification of at least one TLS and categorization by localization. Intra-TLS clusters were defined within the tumor infiltration margins, whereas peri‑TLS clusters were found in normal tissue >10 mm outside the infiltration margins.^20^^,^^30^ This conclusion was made independently by an urologist and two pathologists. According to the spatial location of TLS, samples with at least one intra-TLS are considered intratumoral TLS-positive, while samples with at least one peri‑TLS but no intra-TLS are considered peritumoral TLS-positive.^31^^,^^32^

For TLS maturation analysis, we co-stained slides for CD45, CD20, CD8 and CD23.^33^, ^34^, ^35^ All dense lymphocyte aggregates were subjected to high power field (HPF) image capture, regardless of the signal of the above indicators. Each HPF image was examined to determine TLS maturation status. Mature TLS were characterized by the presence of CD23, indicating the presence of FDC and GC cells aggregation.

Based on the staining intensity and staining distribution, the expression levels of CD8, CD20, CD23 and PD-L1 were scored semi-quantitatively using the immunoreactive score (IRS). Briefly, the IRS = staining intensity (SI) × percentage of positive cells (PP). SI was assigned as follows: 0, negative; 1, weak; 2, mild; and 3, strong. PP was defined as 0 = 0 %; 1 = 0–25 %; 2 = 25 %; –50 %; 3 = 50 %; –75 %; and 4 = 75 %; –100 %. Mean counts of positive CD4 and FOXP3 cells were determined within 5 random fields of view (40×) as counts of Treg, and mean counts of positive peripheral node addressin (PNAD) cells were determined as counts of high endothelial venules (HEVs).

### 2.3 Multispectral fluorescence immunohistochemistry and immunofluorescence staining assays

Multiplex immunohistochemistry (mIHC) was conducted based on our previous research.^11^ To account for the diverse immune components within TLS, we followed established protocols for mIHC. The mIHC procedure utilized the Akoya OPAL Polaris 7-Color Automated IHC Kit (NEL871001KT) with the following panels: Panel 1: CD8, Cytokeratin (CK), PD-L1, DAPI; Panel 2: CD45, CD23, CD20, DAPI; Panel 3: CD4, FOXP3, CK, DAPI. The multiple-stained slides were imaged at 20 nm wavelength intervals from 440 to 780 nm using the Vectra Polaris Quantitative Pathology Imaging System (Akoya Biosciences). A composite image was then generated using inForm software version 2.4.8 (Akoya Biosciences) to quantify the expression of different biomarkers in each cell type.^36^^,^^37^ The number of various cell populations was quantified as the number of stained cells per square millimeter and the percentage of positively stained cells among all nucleated cells, according to the manufacturer's instructions.^38^ The immunofluorescence staining protocol was similar to the IHC staining protocol, with the exception of antigen retrieval. The antibody used for immunofluorescence (IF) staining was anti-PNAD (clone MECA-79, 1:100, BD Pharmingen).

### 2.4 TLS imprint signature and HEV score

To assess the presence of TLS in samples, we employed a transcriptomic data analysis approach based on four well-validated gene signatures. These signatures are specifically designed to detect key genes associated with TLS components, enabling us to determine whether TLS exist within the sample. The Six TLS Indicator signature^39^ comprises six markers (CD19, MS4A1, CXCR5, CXCL13, CCR7, and CCL19), which are critical for the formation and function of B cell and T cell zones within TLS. The Nine TLS Signature^40^ includes nine markers (CCR6, CD79B, PTGDS, and six others) that play roles in antigen presentation, immune signaling, and stromal support. The Twelve-Chemokine Gene Signature^41^ encompasses twelve chemokine molecules (CCL2, CXCL9, and ten others), crucial for guiding immune cell trafficking and positioning within TLS. Lastly, the Twenty-nine Gene Signature^42^ covers a broad spectrum of immune-related functions, including immunoglobulin production and plasma cell differentiation. Molecules such as IGHA1, IGHG1, IL7R, and 25 others highlight diverse cellular elements within TLS, offering comprehensive coverage of TLS-associated processes.

Referencing prior literature,^43^ we selected ten HEV markers: CHST4, CCL21, CCL19, IL33, ICAM1, MADCAM1, TSPAN7, MEOX2, ANKRD53, and ZNF280C and utilized the “AUCell” package^44^ to compute HEV scores based on the spatial transcriptomics data.

### 2.5 Bioinformatics analysis

Somatic mutation data for PCa were retrieved from TCGA database and retained in Mutation Annotation Format (MAF). Mutational analysis was conducted using the R package maftools.^45^ To explore the biological functions and signaling pathways of differentially expressed genes, we performed gene ontology (GO) and Kyoto Encyclopedia of Genes and Genomes (KEGG) enrichment analyses using the R packages clusterProfiler, enrichr, and ggplot2.^46^ We considered conclusions statistically significant when both the *P*-value and FDR threshold were below 0.05. To assess gene set variation across different clusters, we performed Gene Set Variation Analysis (GSVA) using the KEGG gene set (C2, CP, KEGG.v7.2 gene sets), selecting significant results based on |log2-fold change (FC)| > 0.1 and FDR < 0.05. Additionally, C2, CP, KEGG.v7.2 gene sets, and Hallmark collections were obtained from the Molecular Signatures Database (MSigDB) and analyzed using Gene Set Enrichment Analysis (GSEA) software (version 4.0.3), defining highly enriched outcomes as those with an FDR *q*-value < 0.05 and a normalized enrichment score > 0.25. The xCell algorithm was employed to estimate the proportion of immune cell types in PCa.^47^ Survival analysis was carried out using the Kaplan-Meier method, focusing on disease-free survival interval (DFI) and progression-free interval (PFI) as primary endpoints, with critical values determined by the “survminer” package in R and evaluations conducted using the log-rank test with a 95 % confidence interval (CI). DFI refers to the length of time after treatment during which no evidence of disease is found. Commonly used in cancer treatment, it is calculated from the date of treatment completion to the date of disease recurrence or the appearance of new lesions. PFI refers to the length of time during and after treatment that the disease does not worsen. In our study, these data were publicly obtained through the GDC Data Portal.

### 2.6 Spatial multi-omics data analysis

Spatial transcriptome data were obtained both in 10× Genomics official website (https://www.10xgenomics.com/) and Gene Expression Omnibus (GEO, https://www.ncbi.nlm.nih.gov/geo). A total of 18 samples diagnosed as primary PCa tumor (1 from 10× Genomics official website, 17 from GSE278936) were enrolled for following analysis. Combining morphological features and TLS scores, we searched for characteristic TLS structures in the histochemical images of these 18 primary PCa. The management and visualization of spatial transcriptomics data (ST) were carried out using the Seurat R package. To ensure standardization of the ST data, the SCTransform (SCT) method was applied. For integrating the ST data, the functions SelectIntegrationFeatures, PrepSCTIntegration, FindIntegrationAnchors, and IntegrateData were utilized.

Based on the cell type information of single cell analysis in our prior studies,^48^ we conducted deconvolution algorithms. Based on the results of CellTreck, we visualized the cell type component of each TLS. Scoloc_vis function implemented in CellTreck^49^ were used to decipher the colocalization features of each cell. Based on the results from robust cell type decomposition (RCTD) deconvolution algorithyms,^50^ we performed STlearn Cell-Cell communication analysis to further explore the ligand-receptor (L-R) features in TLS. In addition, to further investigated the diverse biological features between different TLS, we also conducted GSEA analysis for filtrated differential expression genes.

We further investigated the correlation between TLS and plasma function, including SDC1, CD138, MZB1, PRDM1, XBP1, IRF4, TNFRSF17, JCHAIN, CD38, SLAMF7, IGHG1, IGHA1, and IGKC. In addition, we compared the differences in Breg and Treg activity between TLS and non-TLS regions, as well as between regions of different maturity.

### 2.7 Statistical analysis

Statistical and graphical analyses were conducted using GraphPad Prism software (version 8.0), R software (version 4.0.2) and python (version 3.9). One-way analysis of variance (ANOVA) was employed to compare differences among multiple groups, while Student’s *t*-test was used for comparisons between two groups. Categorical data were analyzed using the chi-square test. Multivariate Cox proportional hazards regression models were constructed to identify independent prognostic factors for DFI and PFI. Variables included age, race, tumor T stage, N stage, Gleason score, and intratumoral/peritumoral TLS status. Hazard ratios (HR) with 95 % CI were calculated to quantify associations. All covariates entered simultaneously to adjust for confounding effects. Model assumptions were verified via Schoenfeld residuals. All hypothesis tests were two-sided, and a *P*-value < 0.05 was considered statistically significant.

## 3 Results

### 3.1 Baseline demographics and clinicopathological characteristics of the TCGA and FUSCC cohorts

The baseline clinicopathological characteristics of the TCGA (*n* = 501) and FUSCC (*n* = 200) cohorts revealed significant differences (all *P* < 0.001 unless specified) (Table 1). The FUSCC cohort was older (mean age: 65.32 vs. 61.02 years) and comprised entirely Asian patients, while the TCGA cohort was predominantly White (83.03 %). Patients with Gleason scores ≥ 9 were more frequent in FUSCC (51 % vs. 27.35 % in TCGA), reflecting higher tumor aggressiveness. Pathological T4 (17.50 % vs. 2.00 %) and N1 (33.00 % vs. 15.77 %) stages were more prevalent in FUSCC, and 86.50 % of FUSCC patients were classified as high-risk by D’amico criteria compared to 67.66 % in TCGA. Biochemical recurrence was markedly higher in FUSCC (67.00 % vs. 11.58 %). These findings highlight distinct clinical profiles and disease severity between cohorts, potentially influenced by demographic and regional variations.

**Table 1:** Baseline clinicopathological characteristics.

	Mean ± SD or N( %)		
Characteristics	TCGA cohort (*n* = 501)	FUSCC cohort (*n* = 200)	*P*-value
Age, years	61.02 ± 6.82	65.32 ± 7.61	< 0.0001
Race			< 0.0001
White	416 (83.03)	0 (0.00)	
Black or African American	58 (11.58)	0 (0.00)	
Asian	12 (2.39)	200 (100.00)	
Other	1 (0.20)	0 (0.00)	
NA	14 (2.79)	0 (0.00)	
Gleason score			< 0.0001
6	45 (8.98)	7 (3.50)	
7	250 (49.90)	25 (12.50)	
8	65 (12.97)	38 (19.00)	
9	137 (27.35)	102 (51.00)	
10	4 (0.80)	28 (14.00)	
Pathologic T			< 0.0001
T2	188 (37.52)	83 (41.5.00)	
T3	296 (59.09)	64 (32.00)	
T4	10 (2.00)	35 (17.50)	
NA	7 (1.40)	18 (9.00)	
Pathologic N			< 0.0001
N0	349 (69.66)	89 (44.50)	
N1	79 (15.77)	66 (33.00)	
NA	73 (14.57)	45 (22.50)	
D'amico classification			< 0.0001
Low risk	3 (5.99)	1 (0.5)	
Intermediate risk	60 (11.98)	18 (9)	
High risk	339 (67.66)	173 (86.50)	
NA	99 (19.76)	8 (4.00)	
Biochemical recurrence			< 0.0001
No	374 (74.65)	66 (33.00)	
Yes	58 (11.58)	134 (67.00)	
NA	69 (13.77)	0 (0.00)	
TLS			0.248
No	393 (78.44)	158 (79.00)	
Peri-TLS	49 (9.78)	19 (9.50)	
Intra-TLS	87 (17.36)	23 (11.50)	

Abbreviations: FUSCC, Fudan University Shanghai Cancer Center; intra-TLS, intratumoral TLS; NA, not available; Peri-TLS, peritumoral TLS; SD, standard deviation; TCGA, The Cancer Genome Atlas Program; TLS, tertiary lymphoid structures.

Despite these differences in clinical and pathological characteristics, the prevalence of TLS was comparable between the two cohorts, with 22.56 % of patients in the TCGA cohort and 21.00 % in the FUSCC cohort exhibiting the presence of TLS (*P* = 0.248). Overall, while the FUSCC cohort exhibited more aggressive clinical and pathological features compared to the TCGA cohort, the prevalence of TLS remained consistent across both cohorts, underscoring the potential universality of TLS in PCa and its relevance for further investigation into tumor biology and prognosis.

### 3.2 Morphological characterization of TLS in PCa

Our previous studies^11^ demonstrated that HE staining is a highly reliable method in identifying and characterizing TLS. Therefore, analysis of paraffin and frozen sections from the TCGA PCa cohort revealed that TLSs are distinct, cell-dense regions with well-defined organizational features.

**Fig. 1:**
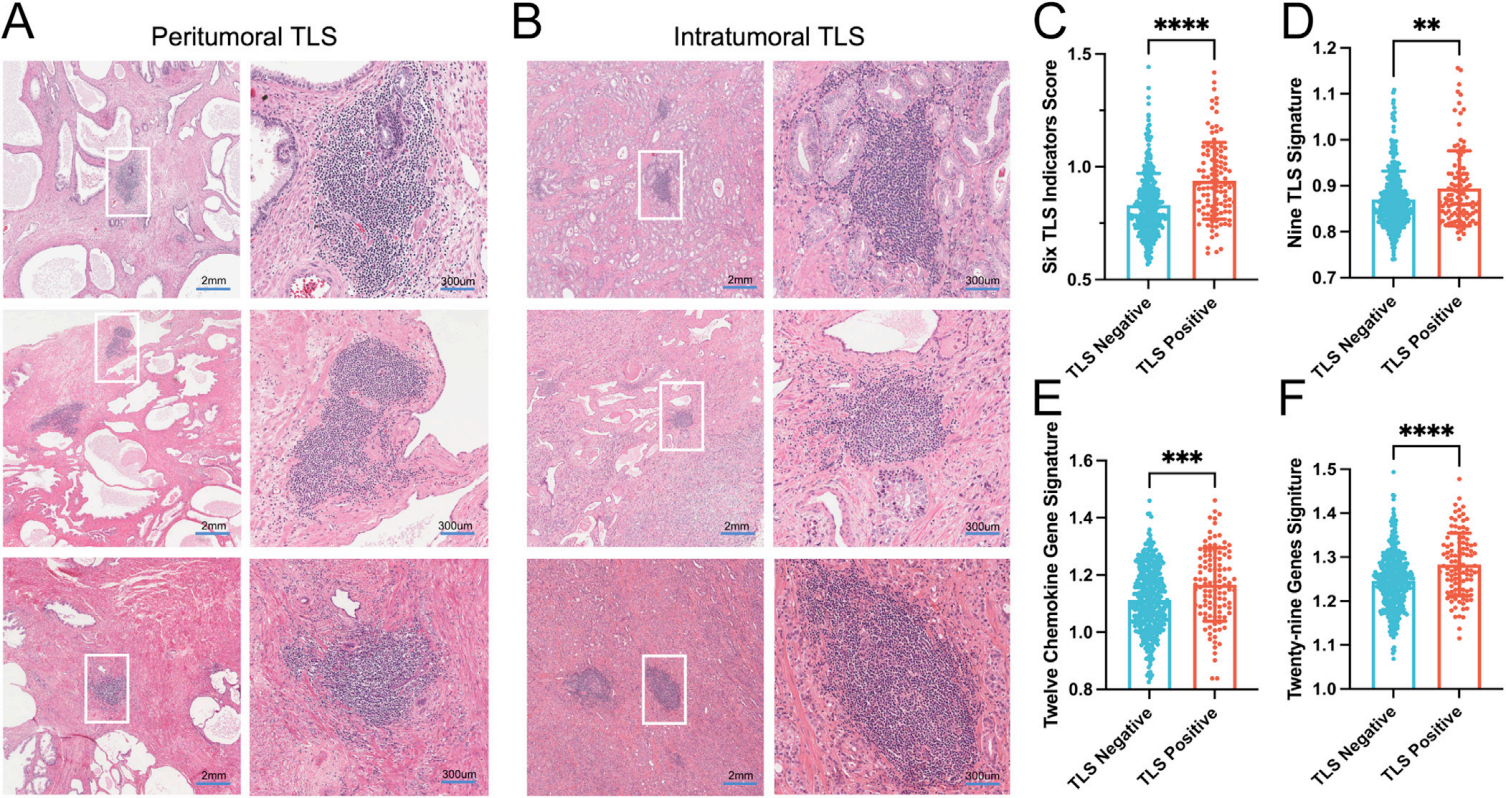
Representative structure of TLS in PCa. (A, B) Representative hematoxylin and eosin staining images of peritumoral (A) and intratumoral (B) TLSs in PCa. Scale bars, (large field) 2 mm; (zoomed in) 300 μm. (C-F) Four TLS algorithm scores for TLS-positive (*n* = 108) and TLS-negative (*n* = 393) patients. ^⁎⁎^, *P* < 0.01; ^⁎⁎⁎^, *P* < 0.001; ^⁎⁎⁎⁎^, *P* < 0.0001. PCa, prostate cancer; TLS, tertiary lymphoid structure.

In HE-stained tissue sections, TLSs were identified as dense lymphoid aggregates exhibiting compartmentalized B-cell follicles and T-cell zones. B-cell follicles appeared as deeply basophilic regions, occasionally containing a centralized dense core suggestive of GC formation. Surrounding these follicles were paler eosinophilic T-cell zones, forming a structured interface indicative of organized immune architecture. Stromal components such as HEVs, characterized by thickened walls and luminal red blood cell accumulation, were frequently observed, supporting TLS functionality in immune cell trafficking. However, while HE staining provided initial morphological insights, distinguishing mature GCs from immature lymphoid clusters proved challenging due to subtle histological differences (Fig. 1A and B). More HE images of intratumoral and peritumoral TLSs were shown in Supplementary Fig. 2. To further validate the presence and functional significance of TLS, we employed four established TLS scoring algorithms to compare TLS-positive and TLS-negative PCa patients.^39^, ^40^, ^41^, ^42^ The results showed that all four algorithms consistently detected significant differences in scores between the two groups of PCa patients (Fig. 1C–F). Overall, these findings underscore the potential of TLS as a key immunological feature in PCa and provide a foundation for further exploration of their prognostic and functional significance.

### 3.3 Association between TLS localization and clinicopathological features in PCa

While previous studies have suggested that the localization of TLS may influence disease progression, the specific relationship between intratumoral and peritumoral TLS and clinical features remains insufficiently investigated across most cancers, including PCa.^31^ In this study, we analyzed the TCGA and FUSCC cohorts by grouping patients based on TLS localization and examined the correlations with clinicopathological characteristics (Supplementary Table 1–10).

Our findings indicated that overall TLS positivity, as well as intra-TLS positivity, is significantly associated with higher pathological T stages in both cohorts. Specifically, in the TCGA cohort, TLS positivity and intra-TLS positivity correlated with higher T stages (*P* = 0.03, *P* < 0.01, respectively), a trend that was similarly observed in the FUSCC cohort (*P* = 0.03, *P* = 0.01, respectively) (Supplementary Table 4 and 9). Furthermore, peritumoral TLS positivity was significantly associated with higher Gleason scores, with this relationship evident in both the TCGA cohort (*P* = 0.04) and the FUSCC cohort (*P* = 0.047) (Supplementary Table 2 and 7).

Interestingly, no significant associations were identified between TLS localization and other clinical features, such as biochemical recurrence, nodal (N) stage or D’amico classification. These results suggested that the localization of TLS, whether intratumoral or peritumoral, may differentially influence specific pathological features of PCa, particularly tumor stage and Gleason score. These findings highlight the importance of TLS spatial distribution in shaping disease characteristics and its potential role as a biomarker for tumor aggressiveness.

### 3.4 Prognostic impact of TLS localization in PCa

The prognostic significance of TLS localization remains a subject of active investigation,^51^^,^^52^ with limited studies addressing its role in PCa. In this study, we specifically evaluated the relationship between TLS localization and clinical outcomes in PCa patients using the TCGA cohort.

**Fig. 2:**
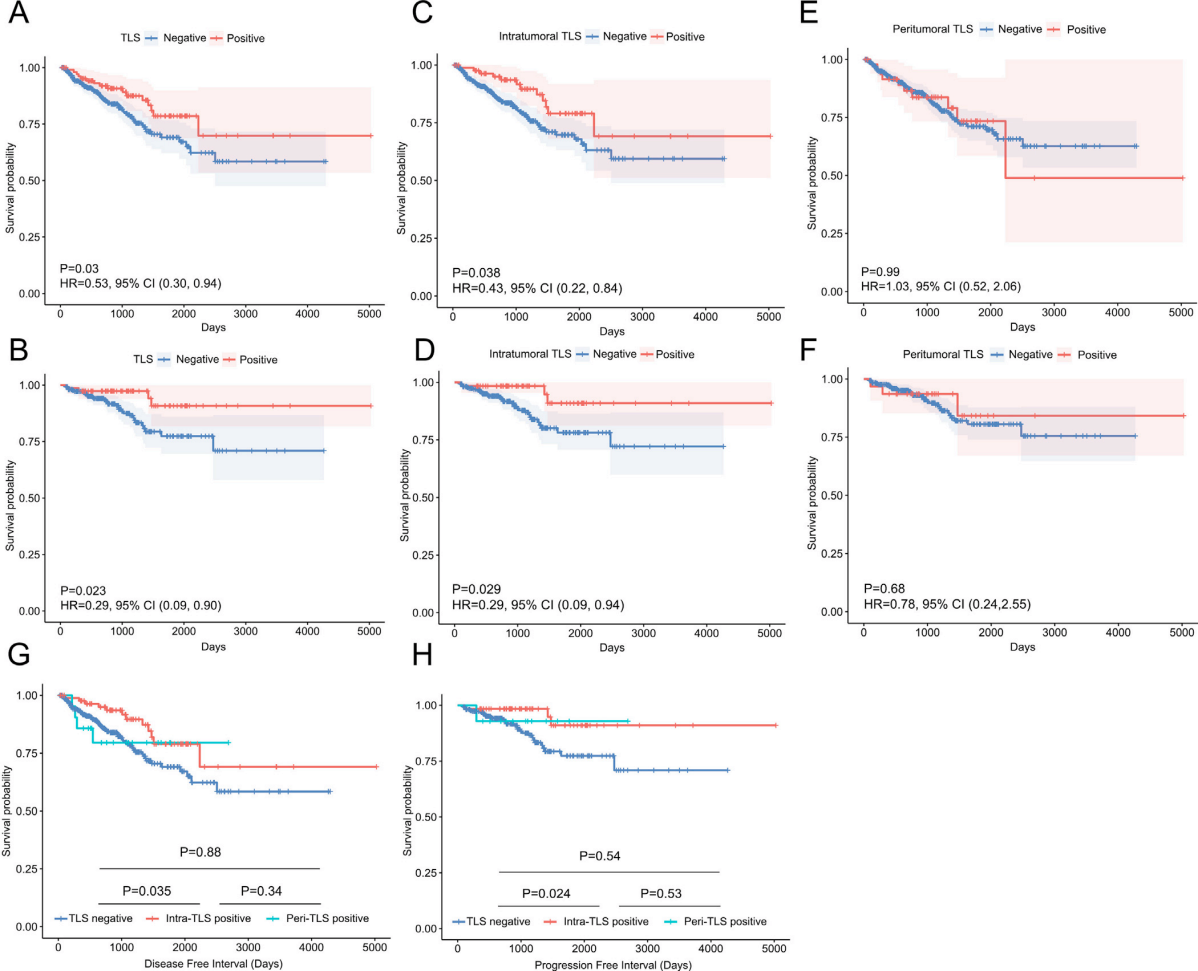
Kaplan–Meier curves for DFI and PFI of three-TLS cluster. (A, B) Kaplan–Meier curves analyzed differences in DFI (A) and PFI (B) for TLS positive and negative PCa patients. (C, D) Kaplan-Meier curves analyzed differences in DFI (C) and PFI (D) for intratumoral TLS positive and negative PCa patients. (E, F) Kaplan-Meier curves analyzed differences in DFI (E) and PFI (F) for peritumoral TLS positive and negative PCa patients. (G, H) Comparative survival analysis of DFI (G) and PFI (H) across intratumoral TLS-positive, peritumoral TLS-positive, and TLS-negative subgroups. DFI, disease-free survival interval; PCa, prostate cancer; PFI, progression-free interval; TLS, tertiary lymphoid structures.

Our analyses revealed that both overall TLS positivity and intra-TLS positivity were significantly associated with improved prognosis, characterized by prolonged DFI and PFI. Specifically, TLS-positive patients exhibited significantly longer DFI (HR = 0.53 [95 % CI, 0.30–0.94]; *P* = 0.030) and PFI (HR = 0.29 [95 % CI, 0.09–0.90]; *P* = 0.023) compared to TLS-negative counterparts (Fig. 2A and B). Similarly, intra-TLS positivity correlated with extended DFI (HR = 0.29 [95 % CI, 0.09–0.94]; *P* = 0.029) and PFI (HR = 0.43 [95 % CI, 0.22–0.84]; *P* = 0.038) (Fig. 2C and D). In contrast, peri‑TLS showed no significant association with either DFI (HR = 1.03 [95 % CI, 0.52–2.06]; *P* = 0.99) or PFI (HR = 0.78 [95 % CI, 0.24–2.55]; *P* = 0.68) (Fig. 2E and F).

Kaplan-Meier survival curves comparing intra-TLS positive, peri‑TLS positive, and TLS negative groups further highlighted distinct survival patterns (Fig. 2G and H). Log-rank tests demonstrated that intra-TLS positive patients had significantly better outcomes than TLS negative patients for both DFI (*P* = 0.0035) and PFI (*P* = 0.0024). Although peri‑TLS positive patients showed a trend toward improved survival compared to TLS-negative individuals, the differences were not statistically significant (DFI, *P* = 0.88; PFI, *P* = 0.54). Notably, intergroup comparisons revealed that intra-TLS positivity conferred a distinct survival advantage over peri‑TLS positivity (DFI, *P* = 0.035; PFI, *P* = 0.024).

The outcomes of the multivariable Cox regression further highlighted the independent prognostic impact of TLS compared to other clinical and molecular factors (Supplementary Fig. 3). The presence of intra-TLS was identified as an independent protective factor, significantly associated with longer DFI (HR = 0.45 [95 % CI, 0.24–0.86]; *P* = 0.015) and PFI (HR = 0.28 [95 % CI, 0.08–0.97], *P* < 0.044). Peri-TLS, while showing a trend toward improved outcomes, did not reach statistical significance after adjusting for other factors (*P* > 0.05).

These findings underscore the potential of intra-TLS as a robust biomarker for favorable prognosis in PCa, while peri‑TLS lacks independent prognostic significance. The spatial localization of TLS thus emerges as a critical determinant of clinical outcomes in this malignancy.

### 3.5 Association between TLS localization and hotspot mutations in PCa

To investigate the relationship between TLS localization and the genomic landscape of PCa, we analyzed the mutational profiles of 20 hotspot genes in the TCGA PCa cohort. Patients were stratified into subgroups based on the presence and localization of TLS, and mutation frequencies were compared across these groups.

**Fig. 3:**
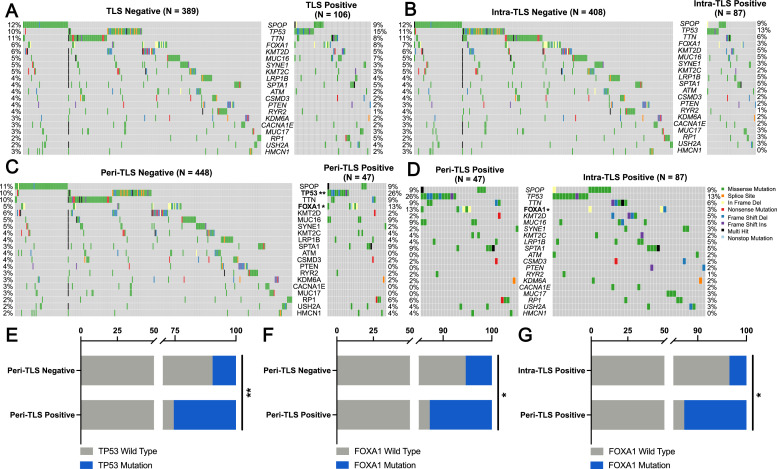
Genomic landscape analysis stratified by TLS spatial localization in PCa. (A-D) Frequency comparison of hotspot mutations between TLS-positive and TLS-negative (A), intra-TLS-positive and intra-TLS-negative (B), peri‑TLS-positive and peri‑TLS-negative (C), intra-TLS-positive and peri‑TLS-positive (D) subgroups. (E-G) Subtype-specific mutation analysis of *TP53* (E) and *FOXA1* (F) in peri‑TLS-positive/negative tumors, and *FOXA1* in intra-/peri‑TLS-positive tumors (G). Data from the TCGA prostate cancer cohort; statistical comparisons performed using chi-square tests, *P < 0.05; ^⁎⁎^*P* < 0.01. PCa, prostate cancer; TLS, tertiary lymphoid structures.

Overall, TLS-positive and intra-TLS-positive patients exhibited slightly lower frequencies of Speckle-type POZ (SPOP) mutations (12 % vs. 9 %; 12 % vs. 9 %) and slightly higher frequencies of *TP53* mutations (10 % vs. 15 %; 11 % vs. 13 %) compared to TLS-negative and intra-TLS-negative patients. However, these differences were not statistically significant (SPOP: *P* = 0.301, *P* = 0.455; *TP53: P* = 0.141, *P* = 0.138) (Fig. 3A and B). In contrast, peritumoral TLS-positive patients demonstrated a distinct mutational profile compared to peri‑TLS-negative patients (Fig. 3C), while revealed differential mutation landscape compared to intra-TLS-positive patients (Fig. 3D). Specifically, peri‑TLS-positive patients showed significantly higher frequencies of *TP53* (*P* = 0.001) and *FOXA1* (*P* = 0.027) mutations than peri‑TLS-negative patients. Furthermore, peri‑TLS-positive patients exhibited a higher frequency of *FOXA1* mutations compared to intra-TLS-positive patients (*P* = 0.040) (Fig. 3E–G). These findings highlight that TLS localization is associated with distinct mutational patterns in PCa. While no significant differences were observed for SPOP or *TP53* mutations in TLS-positive and intra-TLS-positive patients, peri‑TLS-positive patients exhibited a higher frequency of oncogenic mutations, particularly in *TP53* and *FOXA1*. This suggests that peritumoral TLS may be linked to specific genomic alterations, potentially influencing tumor progression in PCa.

### 3.6 Relationship between TLS spatial localization, immune infiltration, and disease progression in PCa

**Fig. 4:**
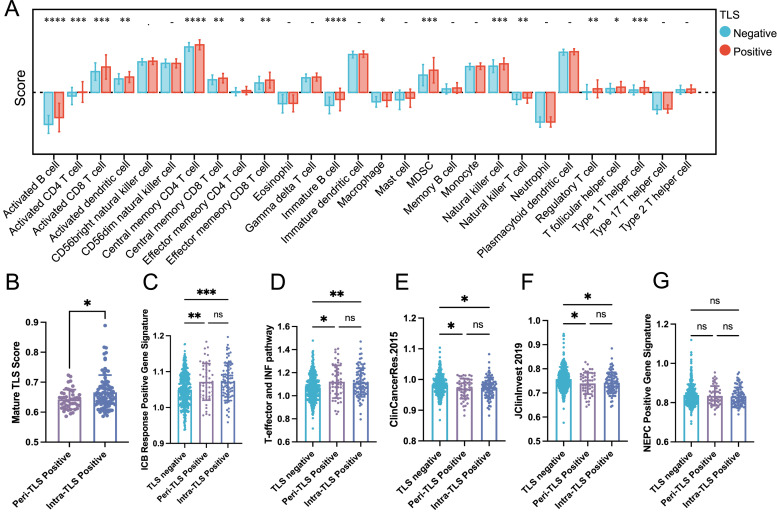
Relationship of TLS localization to immune infiltration and disease progression. (A) xCell algorithm was used to detect differences in immune cell infiltration in different TLS clusters. (B-G) Various algorithms were used to detect onco-immunology pathways in different TLS clusters. *P < 0.05; ^⁎⁎^*P* < 0.01; ^⁎⁎⁎^*P* < 0.001; ^⁎⁎⁎⁎^*P* < 0.0001. ns, not significant; TLS, tertiary lymphoid structure.

Although PCa is typically considered a “cold tumor” with limited responsiveness to immunotherapy, the presence of TLS led us to explore their relationship with immune infiltration and tumor progression. Infiltrating immune cells analysis of the TCGA cohort’s transcriptome sequencing data showed that patients with overall TLS positivity, as well as those with intratumoral or peritumoral TLS positivity, exhibited significantly higher infiltration of activated B cells, CD4^+^ T cells, and CD8^+^ T cells within the tumor tissue, suggesting an enriched immune microenvironment in TLS-positive cases (Fig. 4A and Supplementary Fig. 4).

Further subgroup analysis revealed distinct immune profiles associated with TLS localization. Intra-TLS-positive patients demonstrated an increased proportion of activated DCs, which are critical mediators of antigen presentation and immune activation. On the other hand, peri‑TLS-positive patients exhibited a higher frequency of Tregs, which are known to suppress immune responses and contribute to an immunosuppressive tumor microenvironment. To investigate functional differences between intra-TLS and peri‑TLS, we analyzed TLS maturity scores. Intra-TLS structures were found to have significantly higher maturity scores compared to peri‑TLS (Fig. 4B), suggesting that intra-TLSs are more likely to be fully developed and functionally active immune niches.

In addition, both intratumoral and peritumoral TLS-positive patients displayed higher immunotherapy response scores (Fig. 4C and D), indicating a potential association between TLS presence and enhanced sensitivity to immune-based therapies. Moreover, these patients exhibited lower scores for markers associated with castration-resistant PCa progression (Fig. 4E and F), suggesting that TLS may play a protective role in slowing disease progression. However, no significant differences were observed in neuroendocrine prostate cancer (NEPC) differentiation risk scores across the TLS subgroups (Fig. 4G). Together, these results highlight the potential of TLS to modulate the tumor microenvironment and impact therapeutic responses and disease progression in PCa.

### 3.7 Enrichment and correlation analyses reveal the functional significance of TLS in PCa

To investigate the functional significance of TLS in PCa, we performed pathway enrichment and correlation analyses comparing TLS-positive and TLS-negative patients based on the TCGA cohort’s transcriptome sequencing data. The results revealed striking differences in pathway activity, immune signaling, and metabolic interactions associated with TLS presence and localization. GSVA demonstrated that TLS-positive patients exhibited heightened activity in immune-related pathways and DNA damage repair pathways, accompanied by reduced activity in oncogenic pathways, including WNT, KRAS, and epithelial-mesenchymal transition (EMT) pathways (Supplementary Fig. 5A). Furthermore, intra-TLS-positive patients displayed higher immune pathway activity compared to peri‑TLS-positive patients, suggesting that the localization of TLS influences their functional impact on the tumor microenvironment. To further delineate the biological processes associated with TLS, KEGG and GO enrichment analyses were conducted. These analyses highlighted the enrichment of pathways related to T cell and B cell activation, as well as increased interactions of immune receptor-ligand pairs in TLS-positive patients (Supplementary Fig. 5B and C). These findings underscore the role of TLS in orchestrating adaptive immune responses within the tumor microenvironment.

HEVs, critical structures within TLS, are known to mediate immune cell trafficking by facilitating cell rolling, adhesion, and extravasation into the tumor microenvironment. Functional enrichment analysis further confirmed the activation of the cell adhesion molecule signaling pathway in TLS-positive patients (Supplementary Fig. 5B and C), underscoring the importance of HEVs in promoting immune cell infiltration and facilitating cancer immunity. Finally, correlation analysis between metabolism-related pathways and TLS revealed a positive association between TLS presence and galactose metabolism (Supplementary Fig. 5D). This suggests that TLS may influence or respond to specific metabolic processes within the tumor microenvironment, potentially contributing to the unique immune and metabolic dynamics of TLS-positive PCa. These findings provide insights into the functional role of TLS in shaping the tumor microenvironment and highlight their potential as modulators of immune responses and tumor progression of PCa.

### 3.8 Characterization of maturation heterogeneity of TLS in PCa by mIHC

PCa-associated TLSs were characterized based on their maturation state, with previous studies defining immature TLS as those lacking CD23^+^ germinal center GC cells and mature TLS as those containing CD23^+^ GC cells.^11^^,^^30^ To validate these distinctions, we conducted IHC and mIHC analyses on pathological specimens from the FUSCC cohort, providing insights into the structural and functional diversity of TLS in PCa.

**Fig. 5:**
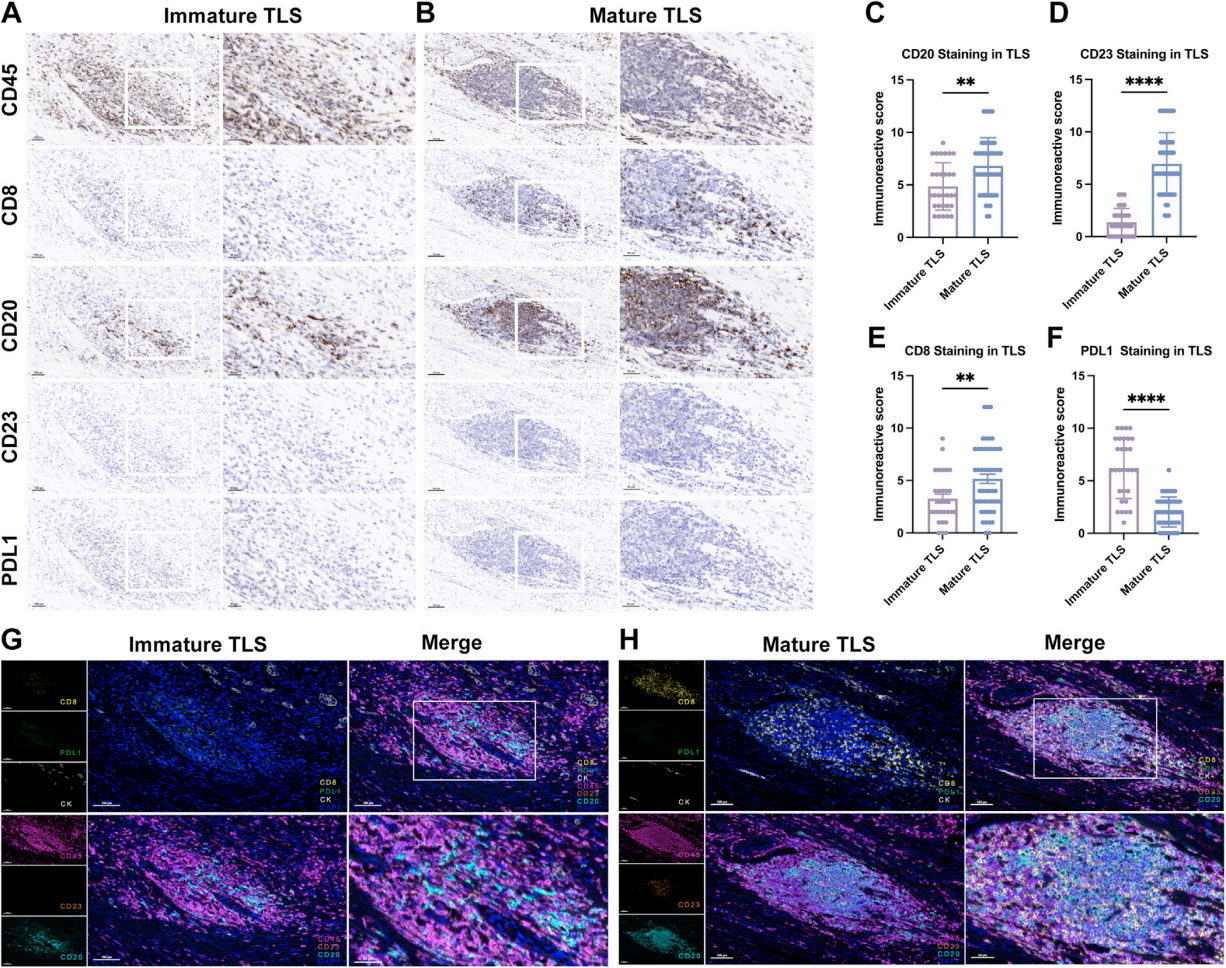
Characterization of mature and immature TLS in PCa by IHC and mIHC. (A, B) Representative IHC staining images of immature TLS (A) and mature TLS (B) in PCa combining CD45 (for immune cells), CD8 (for CD8^+^ T cells), CD20 (for CD20^+^ follicular B cells) and CD23 (for CD23^+^ germinal center cells). Scale bars, (large field) 100 μm; (zoomed in) 20 μm. (C-F) Analysis of immunohistochemical scores for CD20 (C), CD23 (D), CD8 (E) and PD-L1 (F) in mature versus immature TLS. Representative mIHC staining images of PCa tissues depicting the differences in tumor-infiltrated lymphocytes among immature TLS (G) and mature TLS (H). Scale bars, (large field) 100 μm; (zoomed in) 50 μm. *P < 0.05; ^⁎⁎^*P* < 0.01; ^⁎⁎⁎^*P* < 0.001; ^⁎⁎⁎⁎^*P* < 0.0001. IHC, immunohistochemical; mIHC, multiplex-IHC; PCa, prostate cancer; TLS, tertiary lymphoid structure.

IHC staining revealed that mature TLS displayed a highly organized architecture, including distinct regions for B and T cells. CD20^+^ B cells were localized to the inner regions, forming follicle-like structures surrounded by T cells, akin to the organization of secondary lymphoid organs. The presence of FDCs and GC cells within these structures further corroborated their mature status, highlighting their potential role in orchestrating adaptive immune responses (Fig. 5A and B). In contrast, peri‑TLS regions exhibited distinct immunological profiles. Semi-quantitative IHC analysis demonstrated significantly higher PD-L1 IRS scores in mature TLS regions than those in immature TLS regions. Similarly, for CD8, CD20 and CD23, the IRS scores in mature TLS regions markedly exceeded those in peri‑TLS regions (Fig. 5C–F). This finding aligns with the immune infiltration data, which showed a higher proportion of Tregs in peri‑TLS-positive regions, suggesting a more immunosuppressive microenvironment around peri‑TLS.

To further dissect the cellular diversity and spatial organization of TLS at different maturation stages, we performed mIHC using two four-color panels. Panel 1 included CD8, CK, PD-L1, and DAPI, while Panel 2 included CD45, CD23, CD20, and DAPI. This multiplex approach enabled the detailed visualization and quantification of immune cell populations within TLS.

The analysis revealed significant differences in cellular components between immature and mature TLS, as illustrated in Fig. 5G and H. Mature TLSs were enriched with CD20^+^ B cells, CD23^+^ GC cells, and CD8^+^ T cells, indicating a well-developed immune architecture capable of mounting effective immune responses. Conversely, immature TLSs were characterized by a lower density of these immune components and displayed a microenvironment enriched in immunosuppressive features, such as elevated PD-L1 expression, further supporting their limited functional capacity. Overall, mature TLSs exhibit a well-organized immune architecture associated with adaptive immune activation, while immature TLSs are surrounded by an immunosuppressive microenvironment. These findings enhance our understanding of the complex role of TLS in shaping the tumor microenvironment and their potential impact on tumor immunity and progression in PCa.

### 3.9 Impact of TLS maturation heterogeneity on the immune landscape of the TME in PCa

**Fig. 6:**
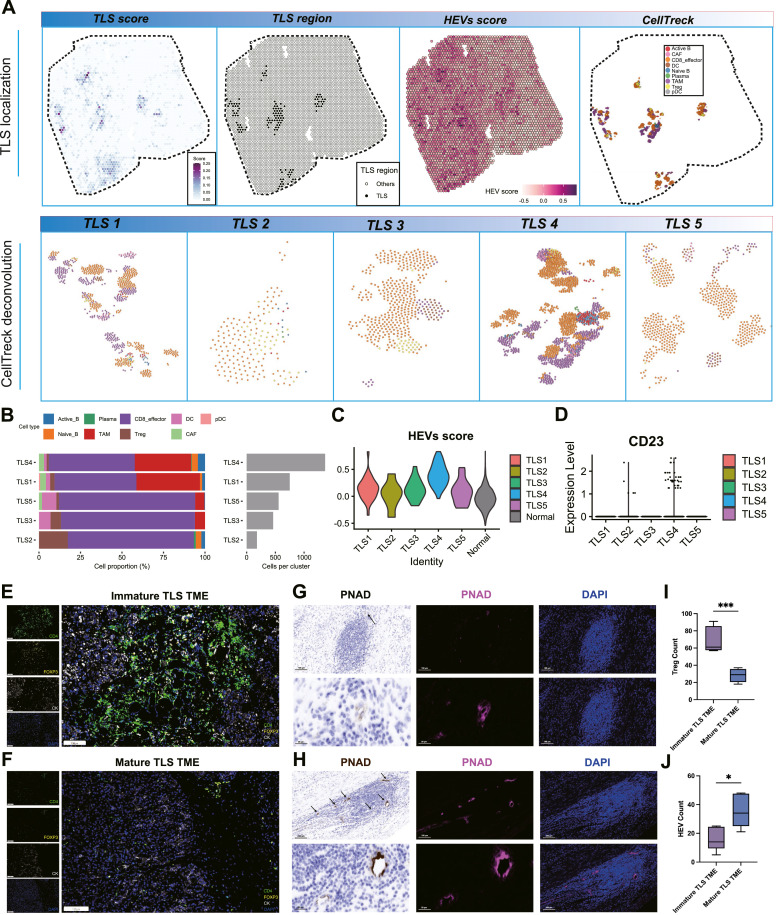
Effects of maturation heterogeneity of TLS on TME in PCa. (A) Spatial plot showed the distribution of TLS scores, TLS regions, distribution of HEVs scores, and cell types. (B) The relative abundance or composition of immune cells within distinct TLS regions (left), and the absolute number of cells in different TLS regions. (C) Comparison of HEVs scores among five TLS positive regions and TLS negative regions. (D) Comparison of CD23 expression among five TLS positive regions. (E, F) Representative mIHC staining images of PCa tissues depicting the differences in TME among immature TLS (E) and mature (F) TLS. (G, H) Detection of PNAD expression levels in immature (G) and mature (H) TLS using IHC and immunofluorescence. Scale bars, (large field) 100 μm, (zoomed in) 20 μm. (I, J) Semi-quantitative analysis of various biomarkers in immature (*n* = 30) and mature (*n* = 45) TLS TME was performed to analyze the percentage of DAPI-positive cells of a specific cell type. *P < 0.05; ^⁎⁎⁎^*P* < 0.001. CAF, cancer-associated fibroblast; DC, dendritic cell; HEV, high endothelial venule; IHC, immunohistochemical; mIHC, multiplex-IHC; PCa, prostate cancer; pDC, plasmacytoid DC; PNAD, peripheral lymphonode vascular addressin; TAM, tumor-associate macrophage; TLS, tertiary lymphoid structure. TME, tumor microenviroment; Treg, regulatory T cell.

Using a combined approach of histomorphological analysis and TLS scoring, we analyzed 18 spatial transcriptomic samples. Among these, 4/18 samples exhibited TLS. The sample from 10× Genomics exhibited the highest number of TLS, classical morphology, and diverse maturation stages. This sample was selected for in-depth analysis. Notably, the TLS-positive region in this sample exhibited significantly elevated HEVs scores compared to the TLS-negative region, consistent with our earlier findings (Fig. 6A).

To further investigate, we performed CellTrek deconvolution analysis to characterize the cellular composition of each TLS region. Among the five TLS regions, TLS4 displayed enhanced immune cell infiltration, marked by an increased presence of activated B cells and a reduced number of Treg cells (Fig. 6B). Comparative analysis of HEVs scores between TLS-positive and TLS-negative regions revealed that TLS4 had the highest HEVs score (Fig. 6C). Additionally, TLS4 exhibited elevated CD23 gene expression, a hallmark of mature TLS (Fig. 6D). Based on these findings, TLS4 was classified as a mature TLS, while the remaining regions were categorized as immature. These results suggest that TLS maturity is associated with distinct immune cell infiltration patterns, potentially influencing the TME and therapeutic responses. Consistently, higher expression of plasma function molecules was also observed in TLS region, such as SDC1, CD138, MZB1, PRDM1, XBP1, IRF4, TNFRSF17, JCHAIN, CD38, SLAMF7, IGHG1, IGHA1, and IGKC (Supplementary Fig. 6A and B). Meanwhile, mature TLS presented higher expression of these function molecules (Supplementary Fig. 6C and D). In the other side of the spectrum, low Treg and Breg activity were observed in mature TLS regions (Supplementary Fig. 6E and F), suggesting its sound immune function and can better exert anti-tumor immune effects.

To further elucidate the effects of TLS maturation heterogeneity on the TME, we analyzed differences in Tregs and HEVs between mature and immature TLS-positive TMEs. Using a mIHC panel with markers including CD4, FOXP3, CK, and DAPI, we quantified the abundance of CD4^+^ FOXP3^+^ Treg cells in the TME (Fig. 6E and F). Additionally, we employed IHC and immunofluorescence to assess the expression of PNAD a marker of HEVs, within the TME (Fig. 6G and H).

Semiquantitative analyses revealed that mature TLS-positive TMEs had a significantly lower abundance of Tregs and a higher density of HEVs compared to immature TLS-positive TMEs (Fig. 6I and J). These findings underscore the critical role of TLS maturation in shaping the immune landscape of the TME, particularly by promoting an immune-activated microenvironment characterized by reduced immunosuppression and enhanced vascularization.

### 3.10 Cell-cell communication dynamics within mature and immature TLS

**Fig. 7:**
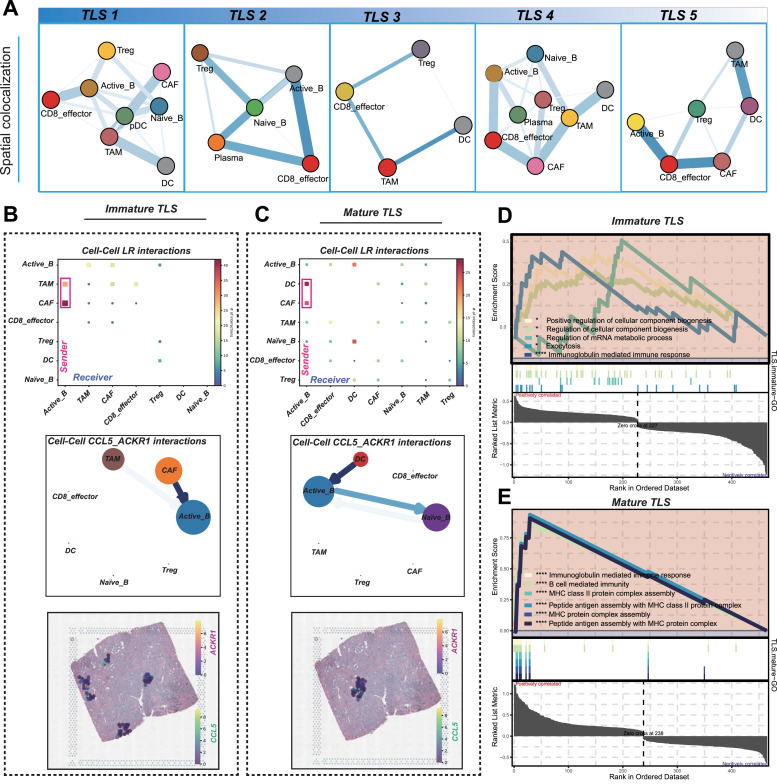
Colocalization identification, cell-cell communication analysis, and GSEA enrichment analysis. (A) Spatial localization of immune components within TLS, the wider the line, the more frequency of colocalization of these cells. (B, C) Total LR interactions (upper), cell-cell interaction network of CCL5 and ACKR1 interactions (middle); colocalization of CCL5 and ACKR1 in spatial image (bottom) among immature TLS (B) and mature (C) TLS. (D, E) GSEA enrichment analysis of immature (D) and mature (E) TLS. *P < 0.05; ^⁎⁎^*P* < 0.01; ^⁎⁎⁎^*P* < 0.001; ^⁎⁎⁎⁎^*P* < 0.0001. CAF, cancer-associated fibroblast; DC, dendritic cell; LR, cell-cell ligand and receptor; pDC, plasmacytoid DC; TAM, tumor-associate macrophage; TLS, tertiary lymphoid structure. Treg, regulatory T cell.

Distinct spatial colocalization patterns of immune cells were observed between mature and immature TLS, revealing significant differences in their cellular interactions and potential functions (Fig. 7A). Specifically, the colocalization frequency of B cells and Treg cells were notably lower in mature TLS compared to immature TLS, suggesting that mature TLS may foster a more effective anti-tumor immune response by reducing immunosuppressive interactions.

To further dissect cellular interactions within TLS regions, cell-cell communication analysis using STlearn was conducted. The resulting interaction networks demonstrated contrasting signaling patterns between mature and immature TLS (Fig. 7B and C). In immature TLS, tumor-associated macrophages (TAMs) and cancer-associated fibroblasts (CAFs) exhibited stronger signaling interactions with activated B cells, potentially contributing to a more immunosuppressive microenvironment. Conversely, in mature TLS, DCs emerged as the dominant mediators of communication with activated B cells, consistent with their role in promoting immune activation.

Chemokine signaling pathways also differed between TLS maturity states. CCL5, an atypical chemokine known to recruit lymphocytes into tumors, was predominantly produced by CAFs and TAMs in immature TLS, binding to the ACKR1 receptor on activated B cells to facilitate their recruitment.^53^ In mature TLS, however, CCL5 production was primarily driven by DCs and CAFs, with both naive and activated B cells interacting to enhance B cell infiltration and TLS development. These findings highlight the heterogeneity in TLS formation and maturation, driven by distinct cell-cell communication networks and chemokine-mediated recruitment processes.

GSEA further revealed that both mature and immature TLS exhibited significant enrichment in the “immunoglobulin-mediated immune response” pathway, underscoring the shared role of TLS in driving antibody-mediated immunity (Fig. 7D and E). However, compared to immature TLS, mature TLS demonstrated markedly higher enrichment in pathways associated with adaptive immune activation, including “B cell-mediated immunity,” “MHC class II protein complex assembly,” and “Peptide antigen assembly with MHC protein complex.” These findings highlight the enhanced capacity of mature TLS to orchestrate robust antigen presentation and B-cell-driven immune responses.

Taken together, this study based on the TCGA and FUSCC cohorts, utilized IHC, mIHC, and spatial multi-omics methods to uncover that mature TLSs in PCa patients are characterized by effector T cell infiltration, mature B cell clustering, increased HEV density, and a rich diversity of immune cells. These features suggest a well-developed anti-tumor immune response and are associated with improved patient outcomes (Supplementary Fig. 7).

## 4 Discussion

TLS have become an intriguing focus in the field of oncology, particularly due to their potential role in enhancing immunotherapy responses.^54^ Numerous studies have investigated the role of TLS in various cancer types and have consistently found that the presence of TLS is positively associated with favorable treatment outcomes.^55^^,^^56^ However, information regarding the role of TLS in PCa remains extremely limited. A more comprehensive analysis and characterization of clinical data on TLS in PCa is warranted. In the present study, we presented several important findings that can serve as a foundation for future research and prognostic prediction.

First, histologic evaluation of TLS revealed its heterogeneity in PCa. Despite differences in baseline clinical characteristics between the two cohorts in this study, the detection rates of TLS were remarkably similar. TLSs are known to arise within the tumor microenvironment under conditions of chronic inflammation and antigenic stimulation. This process may be independent of the clinical stage or severity of the disease, reflecting a generalized immune response to tumor-associated antigens. The prevalence of TLS in PCa further supports their potential as robust prognostic biomarkers, independent of clinical variables such as tumor stage or Gleason score. However, despite comparable prevalence, the functionality of TLS may vary depending on the tumor microenvironment. This functional heterogeneity aligns with emerging evidence indicating that the composition and activity of TLS can differ significantly between patients and across tumor types. We identified both intratumoral and peritumoral TLS in PCa, with a predominance of intra-TLS. Notably, intra-TLS exhibited a higher level of maturation compared to peritumoral TLS. The underlying mechanisms driving these differences in TLS maturation at different locations remain unclear. However, it is hypothesized that the more abundant vascular system within the tumor may contribute to better recruitment of FDCs and lymphocytes in intra-TLS.^57^ Additionally, the presence of an abundant immune component in TLS suggests that patients with TLS may respond better to ICIs treatment.^58^ Therefore, the combination of anti-angiogenic drugs and ICIs in TLS-positive PCa patients represents a promising new direction for future research and therapeutic exploration.

Second, the results of the survival analysis revealed that the localization of TLS is associated with different prognostic trends in PCa. This finding may be related to differences in TLS maturity. Our data indicate that the presence of intra-TLS is associated with a trend toward a better prognosis in PCa, which is consistent with the general perception in other cancer types.^32^^,^^59^ In contrast, the presence of peri‑TLS did not significantly influence the prognosis of PCa. This observation is contrary to what has been reported in some other tumor types. For example, in breast and liver cancers, the presence of peri‑TLS has been associated with a significantly increased risk of recurrence.^17^^,^^31^ Similarly, in renal cancer, peri‑TLS has been linked to shorter OS and PFS.^11^ Additionally, studies in colorectal and lung cancers have emphasized the importance of mature TLS in predicting a favorable prognosis.^20^ Given that intra-TLS, which is predominantly mature, is associated with a better prognosis, it is plausible that TLS maturation, rather than its localization, may be a confounding factor in the observed prognostic differences in PCa. Therefore, further studies on the mechanisms of TLS maturation and their clinical implications are warranted.

Third, recent studies have elucidated the critical role of genetic mutations in the formation and maturation of TLS in cancer. Mutations in DNA damage response genes, such as *TP53* and *BRCA1/2*, are associated with increased genomic instability, which can enhance immune cell recruitment and TLS development.^41^^,^^60^ Conversely, oncogenic mutations, like those in *KRAS* and *BRAF*, can modulate the tumor microenvironment, influencing TLS density and maturity.^37^ Our study revealed that TLS-positive and intra-TLS-positive patients exhibit a lower frequency of SPOP mutations and a higher frequency of *TP53* mutations. This suggests that specific genetic alterations may favor the establishment of an immune-permissive environment conducive to TLS formation. Understanding these genetic-immune interactions is crucial for developing personalized immunotherapeutic strategies, potentially improving patient outcomes.

Forth, mIHC staining further elucidated the differences in the tumor microenvironment between intratumoral and peritumoral TLS in PCa. We observed higher PD-L1 and lower CD8 expression in peritumoral TLS compared to intra-TLS, both of which are indicative of an immunosuppressive environment.^61^^,^ ^62^ Additionally, we found a higher infiltration of Tregs in peri‑TLS than in intra-TLS, a finding that has been associated with adaptive immune resistanc.^61^ When Tregs are selectively recruited to TLS and activated by DCs through antigen presentation, they can suppress DC and T cell function within TLS.^63^ Notably, the depletion of Tregs in mouse models has been shown to trigger the neogenesis of intratumoral HEVs, suggesting that Tregs may inhibit the formation of HEVs and thereby impede the maturation and function of TLS.^42^^,^^64^^,^^65^

Finally, a pivotal strength of our study lies in the exclusive analysis of treatment-naïve prostatectomy specimens, ensuring that the observed associations between TLS characteristics and clinical outcomes are unconfounded by therapy-induced alterations to the TME. Prior therapies—including ADT, radiation, or chemotherapy—are known to profoundly remodel immune landscapes (e.g., ADT-induced CD8^+^ T-cell depletion^66^^,^^67^). By excluding these variables, we captured the *de novo* spatial and functional heterogeneity of TLS in untreated PCa, establishing a baseline for intrinsic TLS biology.

This study, while providing valuable insights into the relationship between tumor-specific TLS and immunotherapy response, has several limitations. The findings are based on retrospective data analysis, and although a potential link between TLS presence and enhanced immunotherapy response was identified, the absence of prospective cohort studies limits the generalizability and robustness of these conclusions. Future research should include well-designed, prospective clinical trials to validate these preliminary observations and further elucidate the role of TLS in predicting and enhancing immunotherapeutic outcomes. Additionally, the current investigation did not fully explore the mechanisms by which the localization and maturation heterogeneity of TLS affect the aberrant TME phenotype in PCa. No in vivo TLS models of PCa were utilized for simulation studies, and future research could benefit from the use of in situ xenograft or patient-derived xenograft models to provide a more detailed understanding of how TLS dynamics impact TME characteristics and therapeutic outcomes. Finally, considering early-stage and advanced PCa exhibit distinct mutational profiles. Our study primarily focuses on localized PCa, where the mutational burden, such as *RB1* and *BRCA1/2*, and microsatellite instability are relatively low compared to advanced or metastatic PCa. Future work will aim to evaluate the landscape of TLS and their role in treatment response in advanced PCa, where mutational burden and immune infiltration are more pronounced.

## 5 Conclusions

In conclusion, this study highlights the prognostic and immunological significance of TLS heterogeneity in PCa. We demonstrate that intra-TLS are more prevalent and exhibit higher maturity compared to peri‑TLS, with mature TLS strongly associated with improved clinical outcomes and a more immunologically active TME. These findings establish TLS maturity and localization as potential biomarkers for favorable prognosis and provide valuable insights into distinct immune phenotypes in PCa. By leveraging TLS heterogeneity, this study offers a foundation for precision patient stratification and the development of tailored immunotherapeutic strategies, with the ultimate goal of improving clinical outcomes in PCa.

## Declaration of competing interest

The authors declare that they have no known competing financial interests or personal relationships that could have appeared to influence the work reported in this paper.

## Appendix Supplementary materials

Image, application 1
