# Supplementary material for: Spatial and maturity heterogeneity of tertiary lymphoid structures shapes immune microenvironment and progression in prostate cancer
Source: J Natl Cancer Cent. 2025 Jul 24;5(5):501–14. doi: 10.1016/j.jncc.2025.06.003 (PMC12529619; doi:10.1016/j.jncc.2025.06.003)

Supplementary materials

Spatial and maturity heterogeneity of tertiary lymphoid structures shapes immune microenvironment and progression in prostate cancer

Zhongyuan Wang, Qintao Ge, Ren Mo, Jiahe Lu, Xi Tian, Aihetaimujiang Anwaier, Shiqi Ye, Siqi Zhou, Weihang Guo, Chuanhai Cai, Jianfeng Yang, Hailiang Zhang, Xiaojian Qin, Dingwei Ye, Wenhao Xu

Supplementary Table 1

The relationship between TLS and BCR in the TCGA cohort.

Group	BCR, No.	Non BCR, No.	OR (95% CI)	*P* value
TLS positive	16	87	1.257 (0.666 to 2.302)	0.472
TLS negative	42	287		
Peri-TLS positive	6	36	1.083 (0.459 to 2.570)	0.863
Peri-TLS negative	52	338		
Intra-TLS positive	10	72	0.749 (0.369 to 1.487)	0.430
Intra-TLS negative	56	302		

Abbreviations: BCR: biochemical recurrence; CI, confidence interval; OR, odd ratio; TCGA, The Cancer Genome Atlas; TLS, tertiary lymphoid structure.

Supplementary Table 2

The relationship between TLS and Gleason Score in the TCGA cohort.

Group	Gleason 8/9/10, No.	Gleason 6/7, No.	OR (95% CI)	*P* value
TLS positive	54	59	1.421 (0.933 to 2.156)	0.102
TLS negative	152	263		
Peri-TLS positive	27	22	1.345 (1.017 to 1.910)	0.036
Peri-TLS negative	179	273		
Intra-TLS positive	44	48	1.398 (0.878 to 2.211)	0.148
Intra-TLS negative	162	247		

Abbreviations: CI, confidence interval; OR, odd ratio; TCGA, The Cancer Genome Atlas; TLS, tertiary lymphoid structure.

Supplementary Table 3

The relationship between TLS and N Stage in the TCGA cohort.

Group	N1, No.	N0, No.	OR (95% CI)	*P* value
TLS positive	21	81	1.198 (0.692 to 2.108)	0.525
TLS negative	58	268		
Peri-TLS positive	13	35	1.767 (0.855 to 3.475)	0.102
Peri-TLS negative	66	314		
Intra-TLS positive	17	64	1.221 (0.682 to 2.226)	0.515
Intra-TLS negative	62	285		

Abbreviations: CI, confidence interval; OR, odd ratio; TCGA, The Cancer Genome Atlas; TLS, tertiary lymphoid structure.

Supplementary Table 4

The relationship between TLS and T Stage in the TCGA cohort.

Group	T3/4, No.	T1/2, No.	OR (95% CI)	*P* value
TLS positive	79	33	1.635 (1.032 to 2.595)	0.0332
TLS negative	227	155		
Peri-TLS positive	33	15	1.314 (0.692 to 2.473)	0.4028
Peri-TLS negative	273	163		
Intra-TLS positive	68	23	2.050 (1.237 to 3.369)	0.0054
Intra-TLS negative	238	165		

Abbreviations: CI, confidence interval; OR, odd ratio; TCGA, The Cancer Genome Atlas; TLS, tertiary lymphoid structure.

Supplementary Table 5

The relationship between TLS and D'amico classification in the TCGA cohort.

Group	High risk, No.	Intermediate/low risk, No.	OR (95% CI)	*P* value
TLS positive	82	10	1.691 (0.852 to 3.430)	0.149
TLS negative	257	53		
Peri-TLS positive	38	5	1.464 (0.577 to 3.564)	0.440
Peri-TLS negative	301	58		
Intra-TLS positive	67	8	1.693 (0.774 to 3.584)	0.1862
Intra-TLS negative	272	55		

Abbreviations: CI, confidence interval; OR, odd ratio; TCGA, The Cancer Genome Atlas; TLS, tertiary lymphoid structure.

Supplementary Table 6

The relationship between TLS and BCR in the FUSCC cohort.

Group	BCR, No.	Non BCR, No.	OR (95% CI)	*P* value
TLS positive	13	29	0.871 (0.434 to 1.791)	0.712
TLS negative	53	103		
Peri-TLS positive	8	11	1.417 (0.543 to 3.719)	0.477
Peri-TLS negative	58	113		
Intra-TLS positive	5	18	0.528 (0.207 to 1.490)	0.222
Intra-TLS negative	61	116		

Abbreviations: BCR: biochemical recurrence; CI, confidence interval; OR, odd ratio; TCGA, The Cancer Genome Atlas; TLS, tertiary lymphoid structure.

Supplementary Table 7

The relationship between TLS and Gleason Score in the FUSCC cohort.

Group	Gleason 8/9/10, No.	Gleason 6/7, No.	OR (95% CI)	*P* value
TLS positive	32	10	0.518 (0.219 to 1.198)	0.120
TLS negative	136	22		
Peri-TLS positive	19	0	Infinity (1.059 to infinity)	0.048
Peri-TLS negative	149	32		
Intra-TLS positive	21	2	2.143 (0.566 to 9.639)	0.476
Intra-TLS negative	147	30		

Abbreviations: CI, confidence interval; FUSCC, Fudan University Shanghai Cancer Center; OR, odd ratio; TLS, tertiary lymphoid structure.

Supplementary Table 8

The relationship between TLS and N Stage in the FUSCC cohort.

Group	N1, No.	N0, No.	OR (95% CI)	*P* value
TLS positive	13	20	0.846 (0.388 to 1.845)	0.677
TLS negative	53	69		
Peri-TLS positive	9	10	1.247 (0.507 to 3.429)	0.652
Peri-TLS negative	57	79		
Intra-TLS positive	12	9	1.975 (0.804 to 4.678)	0.147
Intra-TLS negative	54	80		

Abbreviations: CI, confidence interval; FUSCC, Fudan University Shanghai Cancer Center; OR, odd ratio; TLS, tertiary lymphoid structure.

Supplementary Table 9

The relationship between TLS and T Stage in the FUSCC cohort.

Group	T3/4, No.	T1/2, No.	OR (95% CI)	*P* value
TLS positive	26	11	2.331 (1.054 to 5.065)	0.030
TLS negative	73	72		
Peri-TLS positive	7	6	0.976 (0.346 to 3.112)	0.967
Peri-TLS negative	92	77		
Intra-TLS positive	19	4	4.691 (1.514 to 13.130)	0.007
Intra-TLS negative	80	79		

Abbreviations: CI, confidence interval; FUSCC, Fudan University Shanghai Cancer Center; OR, odd ratio; TLS, tertiary lymphoid structure.

Supplementary Table 10

The relationship between TLS and D'amico classification in the FUSCC cohort.

Group	High risk, No.	Intermediate/low risk, No.	OR (95% CI)	*P* value
TLS positive	35	4	0.951 (0.304 to 2.770)	0.933
TLS negative	138	15		
Peri-TLS positive	17	2	0.926 (0.213 to 4.338)	0.923
Peri-TLS negative	156	17		
Intra-TLS positive	18	2	0.987 (0.230 to 4.606)	0.987
Intra-TLS negative	155	17		

Abbreviations: CI, confidence interval; FUSCC, Fudan University Shanghai Cancer Center; OR, odd ratio; TLS, tertiary lymphoid structure.

Figure legends

Supplementary Fig. 1. Flowchart for selecting the study population in the TCGA and FUSCC cohorts. BCR, biochemical recurrence; FUSCC, Fudan University Shanghai Cancer Center; IHC, immunohistochemical; mIHC, multiplex-IHC; PCa, prostate cancer; TCGA, The Cancer Genome Atlas.

Supplementary Fig. 2. Representative structure of different tertiary lymphoid structure in prostate cancer. Scale bars, (large field) 2 mm; (zoomed in) 300 μm.

Supplementary Fig. 3. Intratumoral TLS associates with prolonged survival in multivariable Cox regression analysis. BCR, biochemical recurrence; CI, confidence interval; HR, hazard ratio; TLS, tertiary lymphoid structure.

Supplementary Fig. 4. Relationship of different TLS clusters to immune infiltration. TLS, tertiary lymphoid structure.

Supplementary Fig. 5. Enrichment analysis and correlation analysis for TLS. (A) Difference in pathway activities scored by GSVA between different TLS clusters. (B, C) KEGG and GO functional enrichment between different TLS clusters. (D) Butterfly plot illustrated the correlation between the TLS and metabolic pathways, the enrichment pathways based on GSVA of GO and KEGG terms. Neg, negative; Pos, positive; TLS, tertiary lymphoid structure.

Supplementary Fig. 6. Comparison of plasma cell function, Treg, and Breg activity across non-TLS, immature TLS and mature TLS regions. (A) Spatial distribution of SDC1, CD138, MZB1, PRDM1, XBP1, IRF4, TNFRSF17, JCHAIN, CD38, SLAMF7, IGHG1, IGHA1, and IGKC. (B) Comparison of Breg function molecules between non-TLS and TLS regions. (C) Comparison of Breg function molecules between immature TLS, mature TLS and non-TLS regions. (D) Comparison of plasma cell function molecules between immature TLS, mature TLS and non-TLS regions. (E) Spatial plot showed the colocalization between TLS regions and Treg and Breg activity. (F) Comparison of Treg and Breg activity among non-TLS, immature TLS and mature TLS regions. Breg, regulatory B cell; TLS, tertiary lymphoid structure; Treg, regulatory T cell. ns, not significant; ****, $P < 0.0001$.

Supplementary Fig .7. Schematic illustration of the study. The study employed HE staining, IHC, mIHC, spatial multi-omics and so forth technologies to examine the spatial and maturation heterogeneity of TLS in PCa. It revealed that intra-tumoral mature TLS contribute to anti-tumor immunity, signifying better prognosis and effective immunotherapy, while immature TLS are associated with poorer outcomes. HE, hematoxylin-eosin; IHC, immunohistochemical; mIHC, multiplex-IHC; PCa, prostate cancer; TLS, tertiary lymphoid structure.

Supplementary Fig. 1

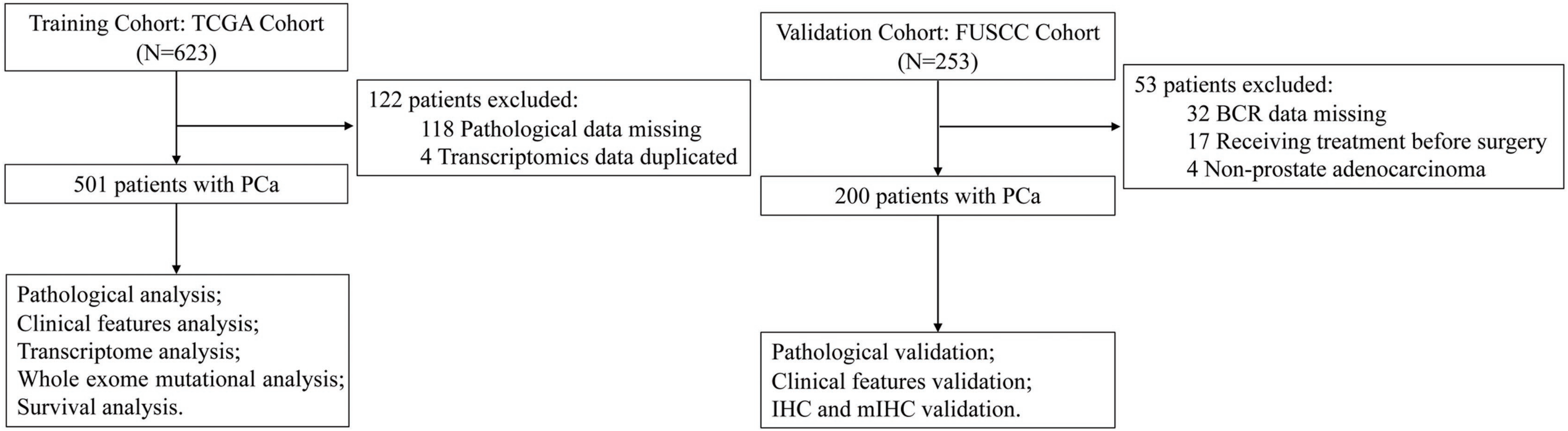

Supplementary Fig. 2

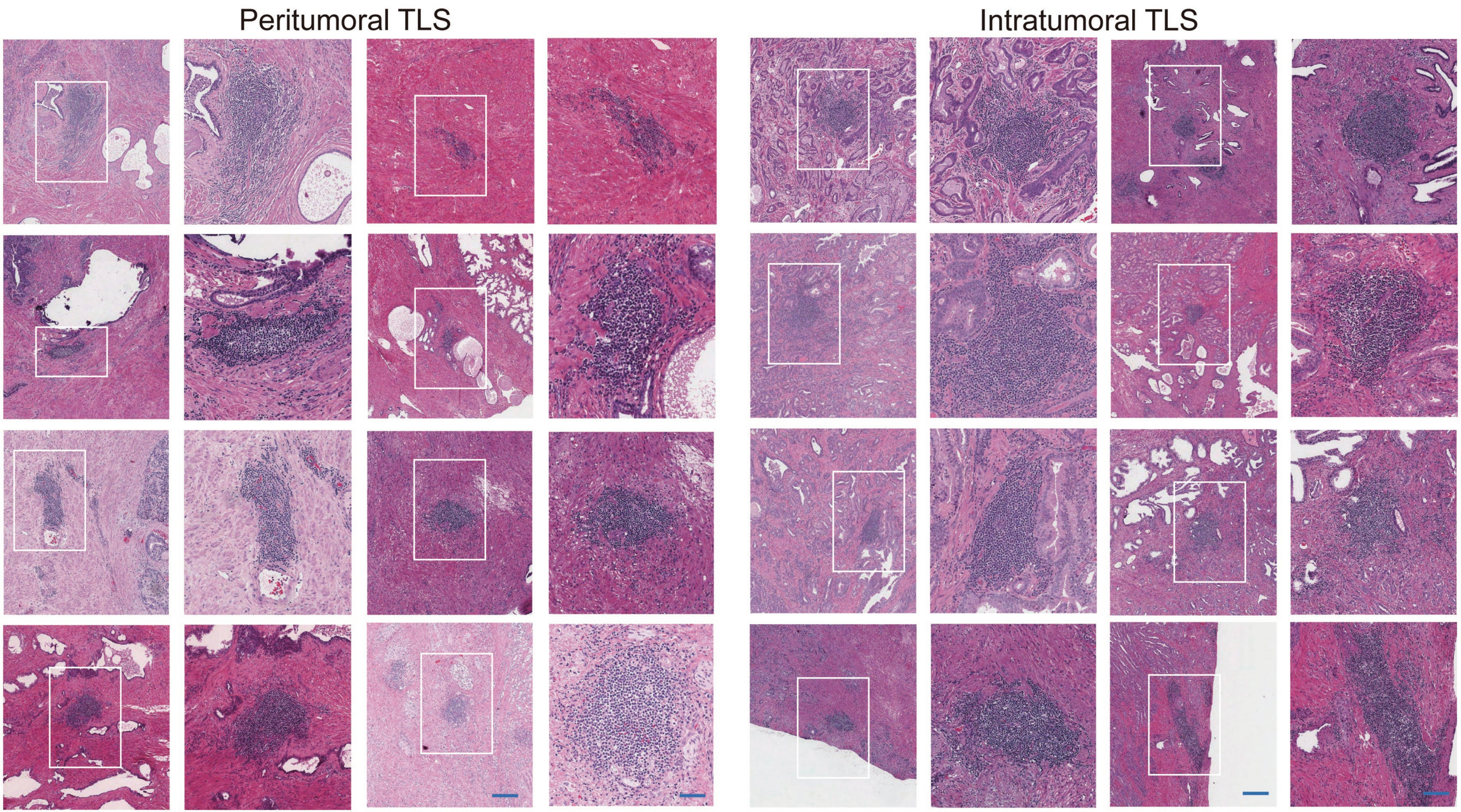

Supplementary Fig. 3

A Disease-free survival interval

Trait	Number	P Value	HR(95% CI)
**TLS**			
TLS Negative (Ref)	393		
Intratumoral TLS positive	87	0.015	0.45(0.24-0.86)
Peritumoral TLS positive	21	0.600	1.33(0.46-3.84)
**Age**			
Age	501	0.677	1.01(0.97-1.04)
**BCR**			
No (Ref)	374		
NA	69	0.513	0.72(0.28-1.90)
Yes	58	0.004	4.35(1.62-11.67)
**Gleason Score**			
Gleason Score	501	0.000	1.76(1.32-2.33)
**Pathologic N**			
N0 (Ref)	349		
NA	73	0.394	0.71(0.32-1.57)
N1	79	0.801	0.89(0.37-2.16)
**Pathologic T**			
T2 (Ref)	188		
NA	7	0.575	0.54(0.07-4.55)
T3	296	0.779	0.74(0.09-5.96)
T4	10	0.754	0.68(0.06-7.39)
**Race**			
Other (Ref)	15		
Asian	12	0.749	0.75(0.13-4.39)
Black or african american	58	0.820	0.83(0.16-4.22)
White	416	0.720	0.77(0.18-3.26)
**D'amico classification**			
Medium (Ref)	60		
High	339	0.793	0.84(0.23-3.08)
Low	3	0.999	0.00(0.00-Inf)
NA	99	0.995	0.00(0.00-Inf)

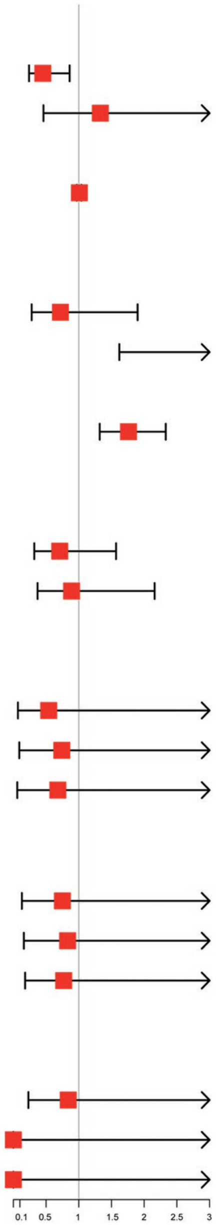

B Progression-free interval

Trait	Number	P Value	HR(95% CI)
**TLS**			
TLS Negative (Ref)	393		
Intratumoral TLS Positive	87	0.044	0.28(0.08-0.97)
Peritumoral TLS Positive	21	0.837	0.81(0.10-6.22)
**Age**			
Age	501	0.057	1.06(1.00-1.13)
**BCR**			
No (Ref)	374		
NA	69	0.970	0.97(0.20-4.62)
Yes	58	0.055	5.47(0.97-30.99)
**Gleason Score**			
Gleason Score	501	0.012	1.86(1.14-3.01)
**Pathologic N**			
N0 (Ref)	349		
NA	73	0.796	0.84(0.23-3.05)
N1	79	0.487	0.56(0.11-2.88)
**Pathologic T**			
T2 (Ref)	188		
NA	7	0.998	9722352.11(0.00-Inf)
T3	296	0.998	18441731.01(0.00-Inf)
T4	10	0.998	16334453.07(0.00-Inf)
**Race**			
Other (Ref)	15		
Asian	12	0.934	0.90(0.08-10.69)
Black or african american	58	0.461	0.33(0.02-6.27)
White	416	0.916	0.89(0.11-7.49)
**D'amico classification**			
Medium (Ref)	60		
High	339	0.466	0.50(0.08-3.19)
Low	3	0.998	0.00(0.00-Inf)
NA	99	0.556	0.59(0.10-3.42)

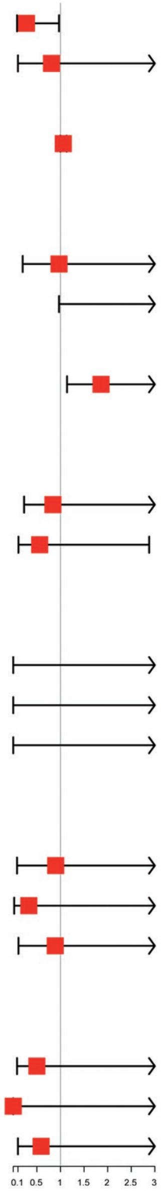

Supplementary Fig. 4

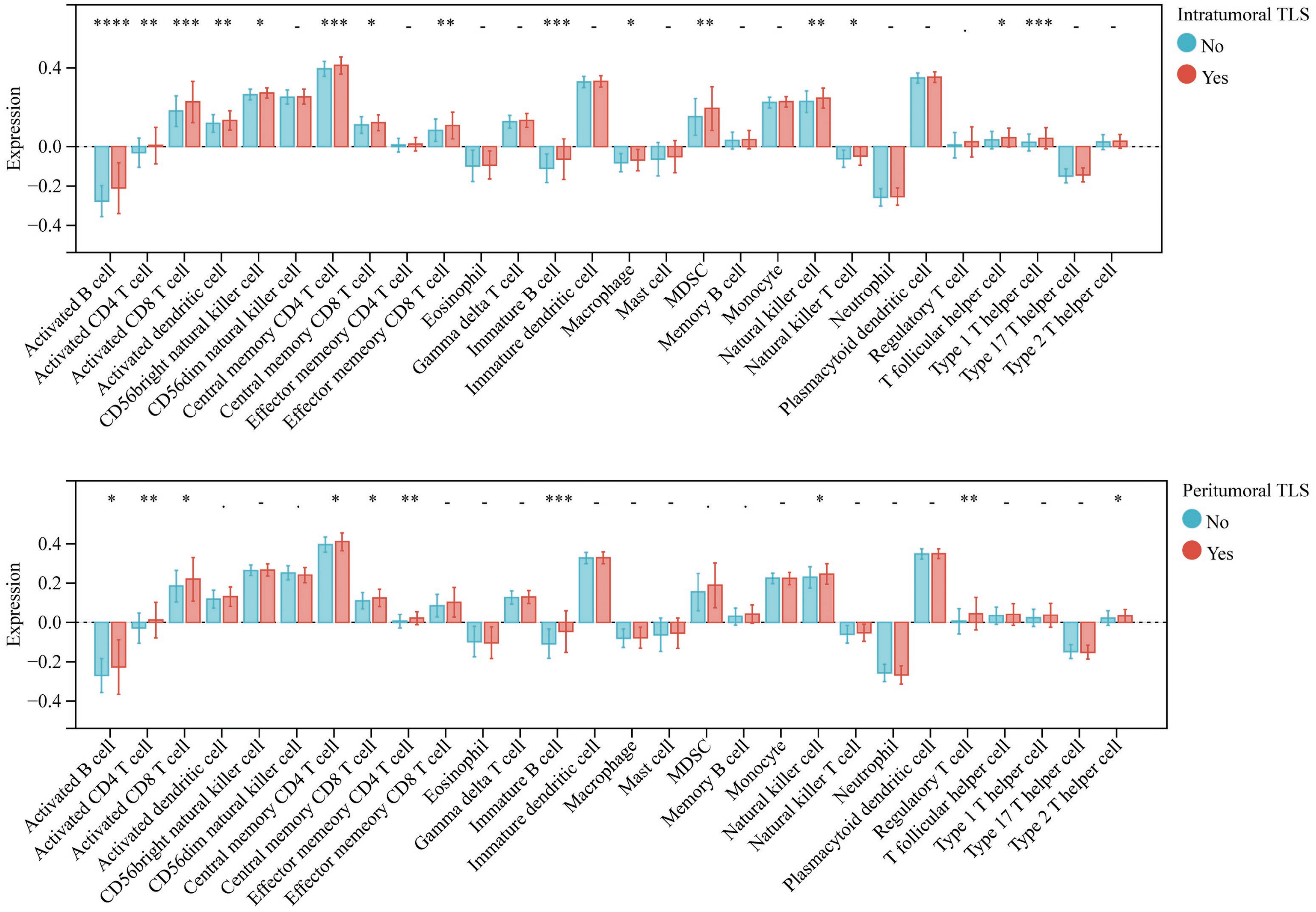

Supplementary Fig. 5

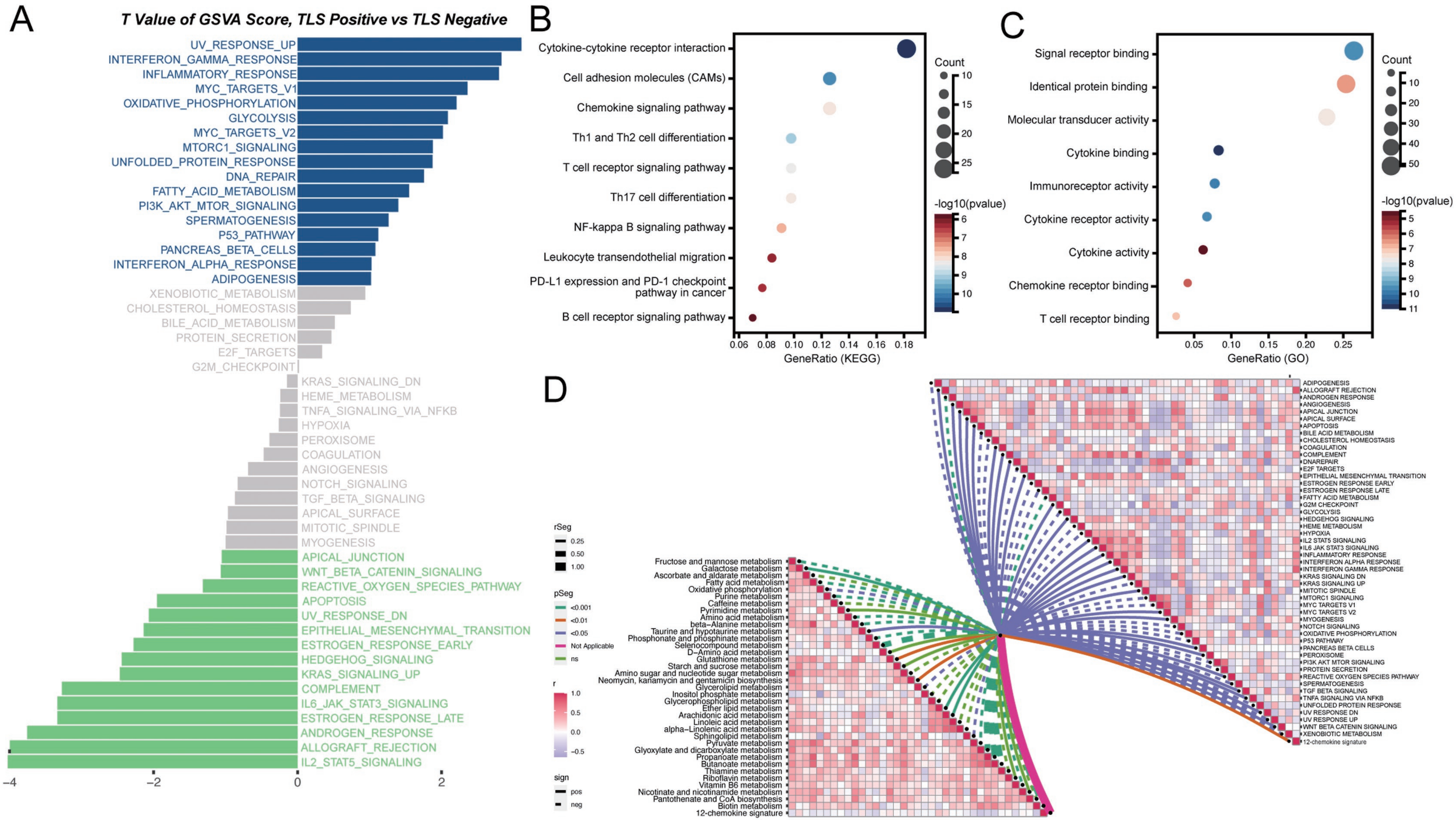

Supplementary Fig. 6

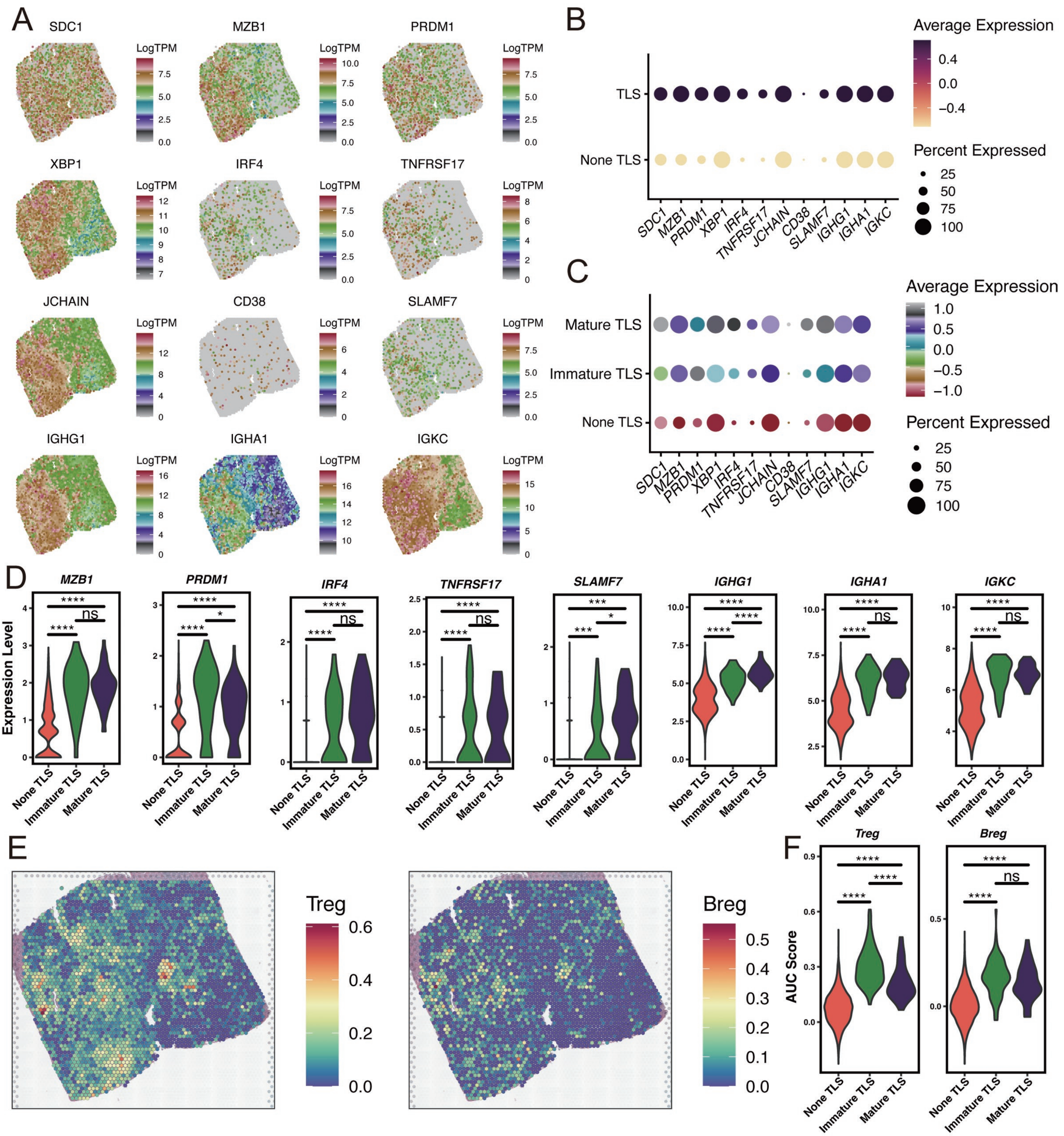

Supplementary Fig. 7

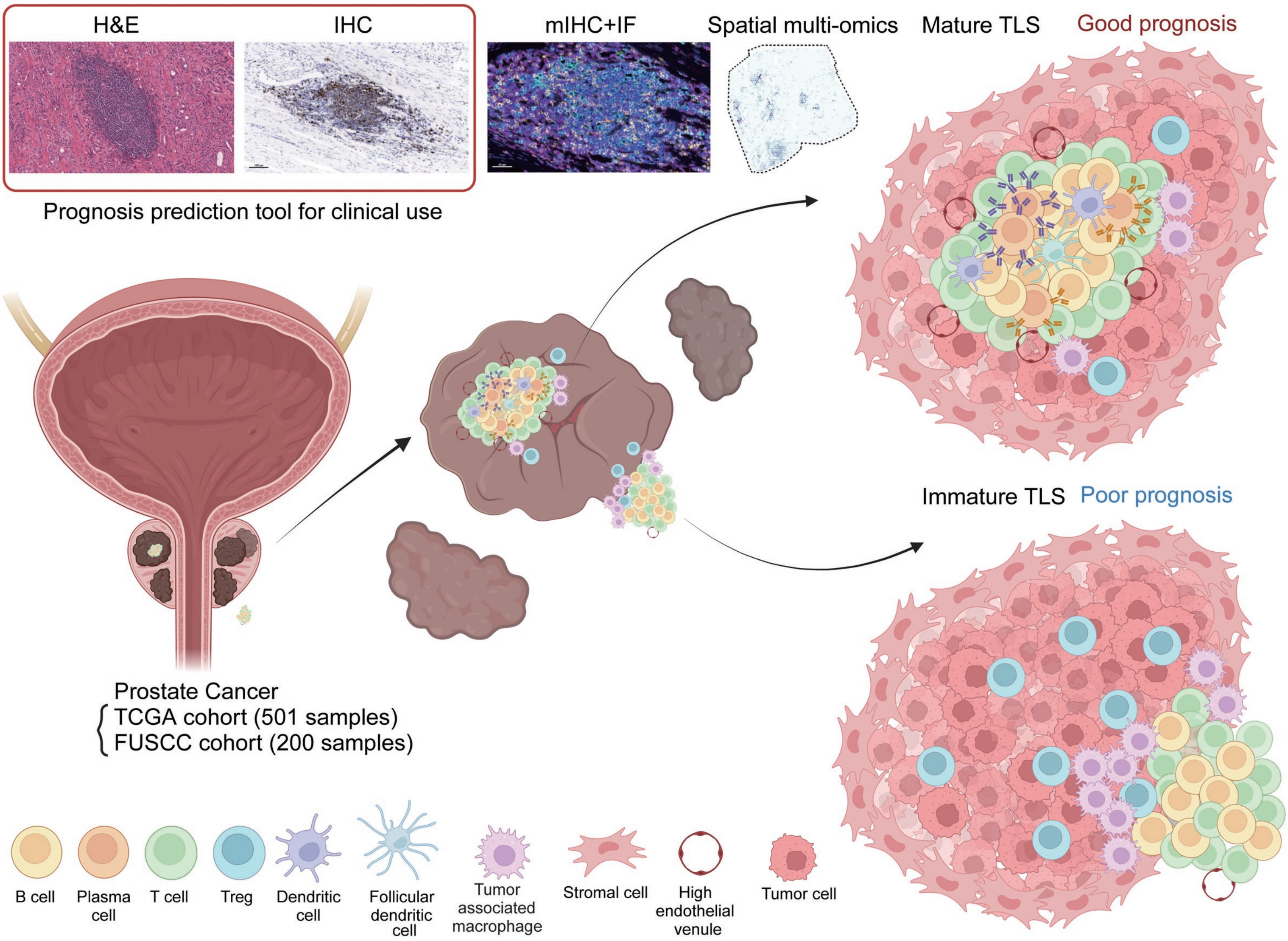

Supplement: Supplementary file 1 [file mmc1.pdf]
